# Determinants of Colour Constancy and the Blue Bias

**DOI:** 10.1177/2041669517739635

**Published:** 2017-12-06

**Authors:** David Weiss, Christoph Witzel, Karl Gegenfurtner

**Affiliations:** Department of Psychology, 9175 Justus-Liebig-University, Giessen, Germany

**Keywords:** adaptation/constancy, categorisation, colour, perception

## Abstract

We investigated several sensory and cognitive determinants of colour constancy across 40 illumination hues. In the first experiment, we measured colour naming for the illumination and for the colour induced by the illumination on the colorimetric grey. Results confirmed that the induced colours are approximately complementary to the colour of the illumination. In the second experiment, we measured colour constancy using achromatic adjustments. Average colour constancy was perfect under the blue daylight illumination and decreased in colour directions away from the blue daylight illumination due to undershooting and a strong blue bias. Apart from this blue bias, colour constancy was not related to illumination discrimination and to chromatic detection measured previously with the same setup and stimuli. We also observed a strong negative relationship between the degree of colour constancy and the consensus of naming the illumination colour. Constancy coincided with a low naming consensus, in particular because bluish illumination colours were sometimes seen as achromatic. Blue bias and category consensus alone explained >68%, and all determinants together explained >94% of the variance of achromatic adjustments. These findings suggest that colour constancy is optimised for blue daylight.

## Introduction

While several important mechanisms and cues to colour constancy are known, it is far from being fully understood (Foster, 2011; Kraft & Brainard, 1999; Smithson, 2005). For example, it is still a matter of debate how colour constancy varies across different illumination colours and whether constancy is particularly tuned to certain illuminations. Here, we evaluated potential determinants that modulate colour constancy in scenes with many densely sampled hue directions.

### Background

It is frequently hypothesised that colour constancy is higher for illuminations varying along the *daylight locus*, where the hue direction of natural illumination varies between yellow and blue over the course of the day. Due to common experience with such colour variation, constancy is expected to be most proficient with changes along the yellow–blue direction (Delahunt & Brainard, 2004; Shepard, 1992).

An alternate hypothesis proposes that the exposure to the frequent variation of colours along the daylight axis produces uncertainty of colour appearance (Beer, Dinca, & MacLeod, 2006; Bosten, Beer, & MacLeod, 2015; Gegenfurtner, Bloj, & Toscani, 2015; Lafer-Sousa, Hermann, & Conway, 2015; Witzel, Valkova, Hansen, & Gegenfurtner, 2011). Several studies observed that bluish illuminations in particular tend to be perceived as neutral, indicating an asymmetry of colour constancy toward the blue direction of the daylight axis that has been called a *blue bias* (Aston, Turner, Le Couteur Bisson, Jordan, & Hurlbert, 2016; Pearce, Crichton, Mackiewicz, Finlayson, & Hurlbert, 2014; Radonjić et al., 2016; Weiss, Witzel, & Gegenfurtner, under review; Winkler, Spillmann, Werner, & Webster, 2015; Wuerger, Hurlbert, & Witzel, 2015). It has been suggested that bluish illumination might be mistaken for shadows (Winkler et al., 2015), which can be bluish in the natural environment due to Rayleigh scattering (Churma, 1994; Troscianko, Benton, Lovell, Tolhurst, & Pizlo, 2009).

Previous findings concerning the relationship between colour constancy and daylight were contradictory. Some studies found evidence for higher colour constancy for blue illumination colours (Daugirdiene, Kulikowski, Murray, & Kelly, 2016; Delahunt & Brainard, 2004), while others did not find differences across illumination hues (Brainard, 1998; Hansen, Walter, & Gegenfurtner, 2007; Olkkonen, Hansen, & Gegenfurtner, 2009; Olkkonen, Witzel, Hansen, & Gegenfurtner, 2010; Schultz, Doerschner, & Maloney, 2006) and some found even better constancy for illumination hues other than blue (de Almeida, Fiadeiro, & Nascimento, 2004; Logvinenko & Tokunaga, 2011). Studies investigating the perception of illumination found that observers had difficulties detecting changes toward bluish illuminations (Aston et al., 2016; Pearce et al., 2014; Radonjić et al., 2016). They suggested that the inability to see an illumination change is an indication of colour constancy. However, one might also make the opposite case and claim that the inability to see an illumination change implies an insensitivity to colour differences, which would undermine colour constancy. Taken together, it is still unclear how colour constancy relates to the variation of daylight.

A third hypothesis to the variation of colour constancy across colours, called *categorical colour constancy*, suggests that colour constancy is related to colour categories. Colour categories are the ensembles of colours designated by colour terms, such as ‘red’, ‘purple’, or ‘blue’. It is yet unknown what determines colour categories and whether and how they are related to colour perception.

Previous research suggests that colour constancy might be highest around the centres of colour categories (Olkkonen et al., 2009; Olkkonen et al., 2010). However, Olkkonen et al. (2009, 2010) did not measure constancy for individual points in colour space, but the constancy of colour categories and their borders across illuminations. Kulikowski and Vaitkevicius (1997) measured colour constancy with an asymmetric matching technique. They found local peaks of colour constancy for typical red, yellow, and blue, and to a lesser extent for green. This finding is substantiated by the observation that surfaces with the prototypical colours of categories have particular physical properties (*sensory singularities*) that make the sensory colour signal more predictable across illumination changes (Philipona & O’Regan, 2006; Vazquez-Corral, O’Regan, Vanrell, & Finlayson, 2012; Witzel, Cinotti, & O’Regan, 2015).

These findings suggest that colour categories developed around the colours that are most stable under illumination changes and hence could serve as ‘perceptual anchors’ under changing illumination (Kulikowski & Vaitkevicius, 1997; Witzel et al., 2015; Witzel, Maule, & Franklin, 2013). The idea of perceptual anchors also fits to a recent observation according to which memorised colours are shifted toward category prototypes (Bae, Olkkonen, Allred, & Flombaum, 2015).

*Relational colour constancy* (Foster & Nascimento, 1994; Foster et al., 1997; Nascimento, de Almeida, Fiadeiro, & Foster, 2004) is another important hypothesis to explain both the variation colour constancy across surface and across illumination colours. According to relational colour constancy, observers use cone ratios to accomplish colour constancy, because these ratios are largely invariant across illumination changes. Predictions based on cone ratios vary depending on the surface colours in a stimulus display and the illuminations, and might explain variation in colour constancy.

More recently, it was suggested that colour constancy is related to *metamer mismatching* (Logvinenko, Funt, Mirzaei, & Tokunaga, 2015; Witzel, van Alphen, Godau, & O’Regan, 2016). Metamer mismatches describe the phenomenon that surfaces that are metameric under one illumination can result in different colours under another illumination (Burns, Cohen, & Kuznetsov, 1989; Cohen & Kappauf, 1982; Logvinenko, Funt, & Godau, 2014; Wyszecki, 1958). Witzel et al. (2016) claimed that higher volume of metamer mismatches (*metamer mismatch volume*) leads to higher uncertainty about a colour under illumination change, and thus weaker colour constancy. They measured colour constancy through asymmetric matching and found a strong relationship between colour constancy and metamer mismatching.

Finally, colour variegation might support colour constancy because the presence of many colours contains information about how changes in illumination affect relations between colours (Golz, 2010; Linnell & Foster, 2002). The colour variegation of a scene may also affect colour constancy through contrast gain control (Brown & MacLeod, 1997) and contrast adaptation (Webster & Mollon, 1995). In particular, a colour that is part of a scene with high colour variegation appears less saturated than the same colour in a scene with low or no colour variegation (see also Ratnasingam & Anderson, 2015; Zaidi, Spehar, & DeBonet, 1997).

### Objective

Taken together, different studies suggest very different determinants of colour constancy across colours, and the question arises how these diverse findings are related. One issue that makes it problematic to compare different studies is that a very limited number of illumination hues were used (mainly four; eight in Brainard, 1998) and that illuminations differed across studies. Some studies investigated illumination hues along and orthogonal to the daylight axis (de Almeida et al., 2004; Delahunt & Brainard, 2004). Others investigated illuminations with colours along the DKL-axes (Hansen et al., 2007; Olkkonen et al., 2009; Olkkonen et al., 2010), which are oriented toward distinctly different hue directions. In particular, the +S endpoint of the so-called blue axis appears purple rather than blue (Malkoc, Kay, & Webster, 2005; Webster, Miyahara, Malkoc, & Raker, 2000; Witzel & Gegenfurtner, 2013, 2015). Another problem that makes comparisons of colour constancy across illumination colours difficult is that the shift of the sensory colour signal due to illumination changes does not just depend on the colour of the illumination, but on the actual spectra of the reflectances and the illuminants. The precise magnitude of the shift due to the illumination is particularly important when evaluating colour constancy through colour constancy indices.

In this study, we tested the candidate determinants of colour constancy across a large number of illumination hues. We used an achromatic adjustment method, which allowed us a high degree of control of experimental conditions (e.g., Brainard, 1998; Speigle & Brainard, 1999). We created two-dimensional variants of the configuration introduced by Lotto and Purves (2002) with illuminations simulated in 40 hue directions. These stimuli are also comparable with regularly arranged Mondrian patterns on a patterned grey-scale background.

This kind of configuration provides a striking illustration of the strong effects of colour induction in rendered scenes under simulated illuminations.

These induction effects correspond to effects of colour constancy: Consider a red surface under a greenish illumination reflecting light that is colorimetrically grey. Due to colour constancy, the colorimetrically grey colour signal is perceived as red, implying that the greenish context of a greenly illuminated scene induces a red appearance. Such strong induction effects also occur for pictures of real objects, as was recently illustrated by Kitaoka (2017). Several studies have shown that colour induction measurements in simulated scenes were largely equivalent to measures for real surfaces and real illuminations, in particular with respect to the variation of colour constancy across colours (Olkkonen et al., 2009; Olkkonen et al., 2010; Radonjić et al., 2016; Speigle & Brainard, 1999). It has been suggested that the daylight axis plays a particular role for colour induction in simple simultaneous contrast displays (Klauke & Wachtler, 2015). Those simple induction effects might be the explanation of the aforementioned effects along the daylight axis observed for colour constancy with realistic scenes.

Using rendered colour in simulated rather than coloured surfaces in real scenes made it possible to collect fine-grained measurements of colour appearance for a large number of hue directions and to control important characteristics of reflectance and illumination spectra. In particular, the large number of directions allowed us to investigate how colour constancy changes as a function of hue, while also enabling statistical comparisons across conditions of interest. To control the effect of the illumination colours on the sensory colour signal, parameters of reflectance and illuminant spectra were matched to produce colorimetric grey in each of the 40 displays. This design also made it possible to investigate the role of illumination colours and the role of the induced colours on the colorimetric grey patches. Finally, we designed the study to match the setup used for the measurement of perceived illuminations in a companion study on the perception of illumination colours (Weiss, Witzel, et al., under review). This allowed us to test in how far the variation of constancy across hue can be explained by how observers perceive the illumination.

In a first experiment, we compared colour categories for illumination colours and the complementary colours induced by the illumination on the colorimetric grey patch. In the second experiment, we measured achromatic adjustments and tested the role of the daylight locus, the blue bias, categorical colour constancy, metamer mismatching, sensory singularities, and relational colour constancy.

## Colour Naming

This experiment provided the colour categories for stimuli in the achromatic adjustment experiment (see below), which allowed us to examine the relationship between colour categories and colour constancy. It has been shown with simple simultaneous contrast displays that the colours induced by simultaneous contrast are complementary, that is, opponent to the inducing colours of the background as predicted by second-stage mechanisms (e.g., Klauke & Wachtler, 2015). However, another recent study (Livitz, Riesen, Shepard, Mingolla, & Eskew, 2016) provided contradictory results. Those previous studies used simple simultaneous contrast displays with two uniform colour areas. This experiment also allowed testing the idea that the colours induced in colour constancy with more complex scenes are opponent to the inducing colours of the illumination.

### Method

#### Observers

Colour naming was measured for 30 German observers (27 women, 22 ± 2years). Observers were students at the Justus-Liebig University as part of an experimental course. All participants were tested for normal colour vision using Ishihara plates (Ishihara, 2004). All experiments were carried out in accordance with the Code of Ethics of the World Medical Association (Declaration of Helsinki) and were approved by the local ethics commission (LEK 2015-0015). Informed consent was obtained from our participants.

#### Apparatus

Stimuli were presented on an EIZO CG2420 monitor driven by an AMD FirePro V4900 with a resolution of 1.920 × 1.200 pixels and a colour resolution of 8 bit per channel. The Monitor was calibrated using a Konika Minolta CS2000 Spectroradiometer (*Konica Minolta* Sensing Inc., Singapore), and CIE-xyY specifications of the channels were: R = [0.685, 0.311, 23.4]; G = [0.216, 0.725, 67.8]; B = [0.151, 0.046, 5.7]. All stimuli used in the experiment have been gamma corrected. The Monitor was placed in a black painted tunnel, 50 cm away from the participant.

The numpad of the keyboard was used for entering responses. The respective keys were marked by the initials of the colour terms, and a printed scheme was also available displaying the complete colour terms in the spatial arrangement of the response keys. Experiments were programmed in MATLAB 2012b (The MathWorks Inc., 2007), using the psychophysics toolbox 3 extensions (Brainard, 1997; Kleiner, Brainard, & Pelli, 2007; Pelli, 1997).

#### Stimuli

**Figure 1.**
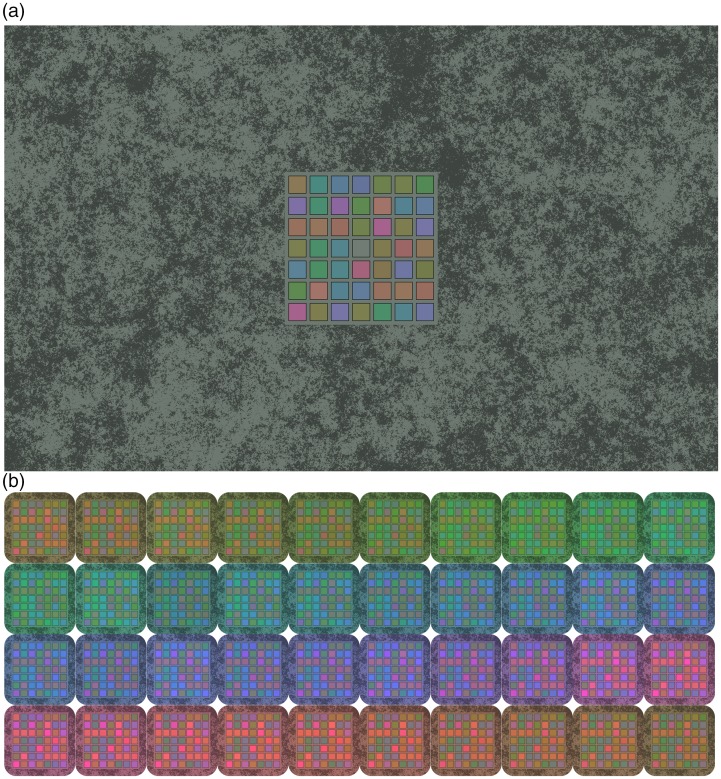
Stimulus display. (a) Scene with background under the neutral illumination. (b) Isolated scene (without background) under each of the 40 chromatic illuminations. For a better overview, only the central checkerboard of the scene is depicted in Panel (b), but in the experiment, all scenes were presented with complete illuminated background as depicted in Panel (a).

Figure 1 illustrates our stimulus display that was inspired by the Purves-Lotto cubes (Figure 9 in Lotto & Purves, 2002). It consisted of a large square (the ‘scene’) composed of 7 × 7 small coloured squares (the ‘patches’) embedded in a background with naturalistic luminance noise (i.e., ‘brown’ noise with an amplitude of 1/f1.7). This display was rendered with a neutral achromatic (Figure 1(a)) and 40 chromatic illuminants (Figure 1(b)).

The particularity of this display is that the test patch in the very centre had the same colorimetric grey colour signal [x = 0.327, y = 0.342, Y = 48.70 cd/m2] under all 41 illuminations. Embedded in a scene under the (simulated) neutral illumination, this colour signal appears grey; but when the (simulated) illumination is chromatic, this same colour signal appears chromatic due to colour induction and colour constancy.

Originally, we designed displays with depth cues that were more similar to the Purves–Lotto cubes.

Preliminary measurements showed that induction effects were similarly strong for the two-dimensional versions without depth cues. Hence, we used the simpler displays in Figure 1 because it simplified the determination of illumination and reflectance spectra.

The challenge in the creation of these displays consists of determining pairs of reflectance and illuminant spectra that all result in the same colorimetric grey, while controlling perceptual parameters of the colours, such as hue and chroma. Another criterion was that we wanted realistic reflectance spectra and smooth illuminant spectra.

To obtain realistic reflectance spectra, we determined the spectra based on the reflectance spectra for matte Munsell chips (Munsell Color Services, 2007). For this, we retrieved the Munsell spectra from the Jeonsuu color group (Kohonen, Parkkinen, & Jaaskelainen, 2006; Parkkinen, Hallikainen, & Jaaskelainen, 1989), which are now available via the University of Eastern Finland (http://www.uef.fi/en/web/spectral/munsell-colors-matt-spectrofotometer-measured). Since these reflectances do not include achromatic reflectance spectra, we used the spectra for Neutral 6.5 and Neutral 5 from the MacBeth ColorChecker (McCamy, Marcus, & Davidson, 1976). These achromatic reflectances were used for the surround with the noise pattern (Figure 1(a)), and the lighter of the two (Neutral 6.5) also defined the colour of the test patch under the neutral illumination. The other 40 reflectances were defined by the 40 Munsell Hues and Munsell Value 7. The latter matched the lightness of the light grey test patch (Colour Checker Neutral 6.5). To control perceived chroma, we linearly interpolated the reflectances for each Munsell hue so that the colour signal resulting of all chromatic reflectances under the neutral illumination (xyY_Judd_ = [0.3265, 0.3419, 136.0]) formed a hue circle in DKL-colour space. The size of the DKL-hue circle was defined by the criterion that the colour signals of all reflectances had to fit into the monitor gamut under all 41 illuminations. Note that the illuminations that shift the colour signal of the equally saturated reflectances to colorimetric grey do not have equal chroma; we come back to this in the experiment on achromatic adjustments (see below).

The coordinates of the DKL-space may be calculated from Judd-corrected Tristimulus Values (XYZ) by the following affine transformation:

$$\begin{array}{c} \text{DKL}={\text{XYZ}}_{\text{Judd}}*\text{T}-\text{S};\\ T=[\begin{array}{ccc} 0 & 0.0188 & 0\\ 0.0342 & -0.0267 & -0.0072\\ 0 & 0.0216 & -0.0216 \end{array}]\\ S=[1 0 0] \end{array}$$

The resulting axes varied between − 1 and 1; for the luminance axis (L + M), this means that − 1 is black and 1 is white.

To obtain smooth illuminant spectra, we created the 41 illuminants based on Gaussian functions. We used a minimisation algorithm to fit the parameters of the Gaussian functions so that the resulting illuminants cancel the colour signal of the complementary Munsell-like reflectance and hence yielded the colorimetrically grey colour signal for that reflectance (xyY_Judd_—coordinates of the illuminants are given in Table S1).

#### Procedure

There were two versions of colour naming. In one version, observers were asked to name the colour of the colorimetrically grey patch in the centre of the display.

This task provided data on how observers categorise the colour appearance induced by context and background based on the simulated illumination. In the second version, observers were asked to name the colour of the background that reflects the colour of the illumination. The presentation of version order was determined randomly.

In both versions, the 41 images were presented one at a time in a random order. A trial began with the presentation of a fixation point for 500 ms, followed by the presentation of the scene until a response was given. Observers could enter a response by pressing one of 11 keys, corresponding to the German Basic Colour Terms: *Rosa* (pink), *Rot* (red), *Orange* (orange), *Gelb* (yellow), *Grün* (green), *Blau* (blue), *Lila* (purple), *Braun* (brown), *Schwarz* (black), *Grau* (grey), and *Weiß* (white).

For each version of the naming task, the complete set of images was presented three times in three consecutive blocks, separated by a short break. Overall, the measurements for both versions took about 15 min.

### Results and Discussion

**Figure 2.**
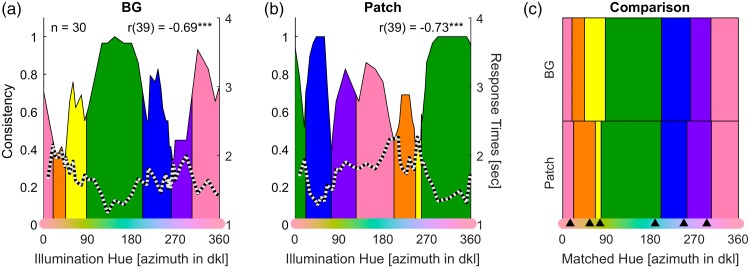
Results from colour naming. (a) Illustration of the colour naming for the colour of the background and (b) the results for naming the induced colours on the test patch in the centre. (c) A comparison of colour naming of the illumination in the background (Panel (a)), the patch with the induced colour (Panel (b)), and colour naming for simple coloured patches as obtained in a previous study (Witzel & Gegenfurtner, 2013). In all panels, the x-axis represents hue of the illumination as determined by azimuth in degree in DKL-space, and coloured areas and vertical lines indicate the mode colour terms and their category boundaries. In the lower part of Panel (c), the azimuth of the illumination has been shifted by 180° in order to approximate the induced hue of the patch so as to match the hue of the illumination and of simple colour patches. In Panels (a) and (b), the left y-axis represents the consensus of colour naming (i.e., the consistency across observers) and the right y-axis the average response times in colour naming. The thin solid curve above the coloured areas and the thin dotted curves in Panels (a) and (b) show the variation of consensus and response times across hues. The correlation between consensus and response times is given in the upper right corner. The black triangles in Panel (c) show the category boundaries for simple colour patches obtained in the previous study (same as in Figure 9(a) in Witzel & Gegenfurtner, 2013). Note the correlation between consensus and response times in both, induced colour (a) and background (b) naming, the high degree of similarity between the categories obtained for induced colours, background colours, and uniform colours (c).

Figure 2 illustrates the aggregated colour categories obtained from the two versions of the colour naming task. To calculate the azimuth, the grey of the neutral background and test surface were used as the origin. For further details, the corresponding individual naming data may be found in Figure S1 of the Supplementary Material. The data in Figure 2 has been aggregated by determining the mode colour term for each stimulus display. Category membership is uncertain at the boundaries and category boundaries are not sharp and clear-cut (e.g., Figure 8 in Olkkonen et al., 2010; Figure 6 in Witzel & Gegenfurtner, 2013; Witzel, Hansen, & Gegenfurtner, 2008). Hence, we determined the boundaries at the hue that had a probability of 50% of being included in one or the adjacent colour category (as in Figure 7 of Witzel & Gegenfurtner, 2013).

#### Category membership

Figure 2(a) illustrates category consensus and average response times for naming the illumination colour reflected off the background. Consistency and average response times are measures of the uncertainty of category membership (Olkkonen et al., 2010; Witzel et al., 2008). With higher uncertainty toward category centres consistencies increase, and response times decrease, and vice versa toward category boundaries. As a result, these measures are negatively correlated (Witzel et al., 2008). Consistency and response times for the background naming in our study are highly correlated across colours, *r*(39) = − 0.73, *p* < .001.

Figure 2(b) shows the category consensus (consistency of naming across observers) and response times for naming the induced colour of the test patch in the centre of the display. As a hue coordinate for the induced colour, the hue opponent to the illumination hue is shown along the x-axis. For the induced colours, consistency and average response times were also highly correlated across the 41 colours, *r*(39) = − 0.69, *p* < .001, indicating that there was a clear consensus of category membership across observers.

Consistency for naming the background was significantly lower than consistency for naming the induced colours of the test patch, *t*(80) = 2.8, *p* = .006. This is noteworthy because the colours in the background were really chromatic while those of the patch are induced colours due to colour constancy. One could have expected induced colours to be more elusive and less consistent, but these results show that this is actually not the case. At the same time, lower consistency for naming of the illumination colours in the background can be explained by partial adaptation to the background colour. Adaptation desaturates the perceived colours and desaturated colours are named with lower consistency (Olkkonen et al., 2010; Witzel, 2016; Witzel et al., 2015).

Another point is noteworthy about the lower consistency of background naming. Consider Figure S1(b). Bluish illuminations were comparatively often described by achromatic colour terms (grey or white). Other hues were never categorised as achromatic. This is in line with the idea of a blue bias in the perception of illuminations as observed previously (Aston et al., 2016; Pearce et al., 2014; Radonjić et al., 2016; Weiss, Witzel, et al., under review). At the same time, bluish induced colours were never described by achromatic colour names when naming the induced colours of the test patch (Figure S1(a)), suggesting that the blue bias is specific to the perception of the illumination.

#### Opponency of induced colours

We examined whether colour categories for induced colours are rotated by 180° in DKL-space compared with the categories of inducing colours. Figure 2(c) allows for comparing the colour categories obtained for the induced and for the inducing colours of the test patch and the background, respectively. In general, categories for induced and inducing colours closely correspond to each other in the upper and lower part of Figure 2(c).

However, there were also differences. The main difference occurred for the yellow category, which is much smaller for patch than for background naming. For each observer, we calculated differences between the boundaries of the two kinds of naming, and calculated t-test across observers to establish whether the differences were significant. It must be noted that this test is subject to additional noise due to the fact that different observers employed different sets of categories, resulting in different kinds of boundaries, for example, brown-green and yellow-green (cf. Figure S1(a) and (b)). We only consider results that are consistent across the two tests for adjacent boundaries (e.g., yellow-green vs. green-yellow). The yellow-orange boundary was significantly different in both tests (both p < .001). The yellow-green, the green-blue, and the purple-pink boundaries were only significant in one of the tests due to occasional grey, brown, and red naming (see Figure S1(a) and (b) for details).

To assess how close these naming sets are to categorisation of simple uniformly coloured patches, we compared them to the categories obtained in a previous study (see Figure 9(a) in Witzel & Gegenfurtner, 2013). Despite slight differences in the white-point (origin of DKL-space), category boundaries for uniformly colored patches (black triangles) were close to those for patch and background naming.

In sum, induced colours are consistently named and show clear category memberships. There was also some evidence for a blue bias for perceived illuminations (Aston et al., 2016; Pearce et al., 2014; Radonjić et al., 2016; Weiss, Witzel, et al., under review). In addition, the hues of induced colours can be roughly approximated by the opposite hue direction in DKL-space. As a result, the hues opponent to the illumination hues may be used as a measure of hue for the appearance of induced colours on the patches. The only exception was the yellow category, which is close to the –S pole of DKL-space. This observation is in line with the observation of Livitz et al. (2016) that induced colours along the S-axis might not be exactly complementary.

The comparatively small yellow category for induced colours may be explained by the idea that blue illuminants are less saturated and weaker inducers. This idea is supported by two instances of grey naming for the orange-yellow colours that are opponent to bluish illuminations (Figure S1(a)). This is in line with the observation that the blue bias in illumination discrimination is related to the anisotropy of colour space that is reflected in the variation of sensitivity across hues (Weiss, Witzel, et al., under review).

## Achromatic Adjustments

In this main experiment, we measured colour constancy through achromatic adjustments for illumination changes in 40 hue directions. We examined how colour constancy changes depending on the hue of the illumination and tested the role of candidate determinates of the variation of colour constancy across hues.

### Method

To compare the results of this experiments to those of the companion study (Weiss, Witzel, et al., under review), the same participants were measured and the same apparatus was used. The 40 stimuli used here included the 12 from that companion study.

#### Observers

Another 16 naïve observers (10 females, 21–31 years old) participated in the achromatic adjustments. All observers were students of the Justus-Liebig University, tested for normal vision using Ishihara plates (Ishihara, 2004) and gave informed consent before participating.

#### Apparatus

Stimuli were presented on an EIZO CG223W monitor driven by an AMD FirePro V4900 with a resolution of 1680 × 1050 pixels, and a colour resolution of 8 bit per channel. The Monitor was calibrated using a Konika Minolta CS2000 Spectroradiometer (*Konica Minolta* Sensing Inc., Singapore), and CIE-xyY specifications of the channels were: R = [0.655, 0.332, 34.6]; G = [0.207, 0.678, 64.2]; B = [0.15, 0.065, 7.8]. All experimental stimuli were gamma corrected. The Monitor was placed in a black painted tunnel, 40 cm away from the participant. From this distance, the screen subtended a visual angle of 61.3° × 40.6°. The distance was fixed by a chin rest mounted to the table. The experiments were programmed in MATLAB 2012b (The MathWorks Inc., 2007), using the psychophysics toolbox 3 extensions (Brainard, 1997; Kleiner et al., 2007; Pelli, 1997).

#### Stimuli and Procedure

The same stimuli were used as in the colour naming experiment (cf. Figure 1). In each trial, observers were presented one of the 41 stimuli and were asked to adjust the test patch in the centre until it appeared achromatic to them. In the instructions, it was emphasised that the test patch should not appear reddish, yellowish, greenish, bluish, or otherwise colourful.

Initially, the test patch had the same colorimetric grey (cf. Figure 1 and Table S1: Neutral) for all 40 chromatic illumination colours. However, to see the test patch as achromatic, observers had to compensate for the induced colour and adjust the patch toward the hue of the illumination, which is opponent to the induced hue (see colour naming experiment above). Observers were not told that the test patch was physically identical across stimuli to avoid cognitive efforts to counteract induction effects. For the control display with the achromatic illumination, the test patch was shown in a random initial colour.

To adjust the colour of the test patch, observers could press one of four keys. The keys corresponded to the four opponent directions of DKL-space and were spatially arranged accordingly. Luminance was fixed to the maximum luminance of the background. There were two different step sizes available, so that the observers could first approximate the colour region they aimed for and then fine-tune their match.

After confirming the adjustments, a sequence of colour noise patterns was presented in order to prevent after images in the following trial (cf. Figure S2). The noise in these sequences changed with every frame and the sequence lasted 3 s.

Each of the 41 scenes was adjusted twice in interleaved order, resulting in overall 82 trials. Before starting the experimental trials, participants performed practice trials until they felt comfortable with the task. A session of adjustment took about 50 minutes.

### Results

#### Patterns of adjustments

**Figure 3.**
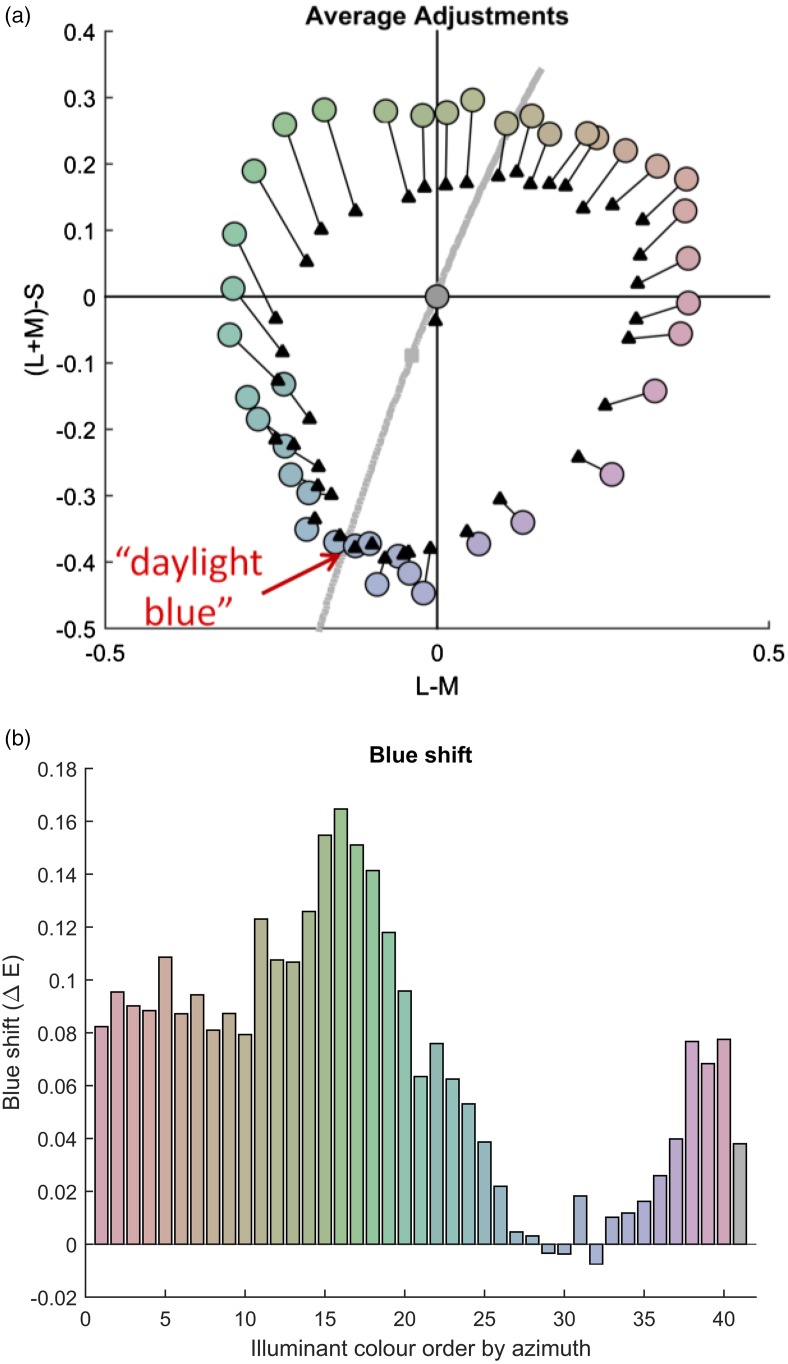
Achromatic adjustments. (a) Illumination colours (coloured disks) and average adjustments (black triangle) in DKL-space, with the L-M contrast along the x- and the (L + M)-S contrast along the y-axis. The grey curve in the background represents the daylight locus; the grey square on the daylight locus corresponds to D65. The red arrow identifies ‘daylight blue’. (b) Illustration of the blue shifts (y-axis) as a function of hue (azimuth along the x-axis). The blue shift quantifies how much closer achromatic matches were to daylight blue as compared with illumination colours. The last bar corresponds to the adjustment in the control condition. Note that almost all achromatic adjustments were shifted toward blue.

Figure 3(a) compares the achromatic adjustments averaged across the 16 observers (black triangles) to the illumination colour (coloured disks). Here, the illumination colour is the colour of the illumination reflected off the grey surface. Individual data may be found in Figure S3.

In contrast to previous studies (Bosten et al., 2015; Chauhan et al., 2014; Witzel et al., 2011), adjustments in the control condition with the neutral illumination did not vary along the daylight axis. Three observers provided strong shifts to the reddish hue direction, while providing sensible measures in the colour constancy conditions. Even when excluding these observers, a variation along the daylight axis was not clearly visible (Figure S3(a)). In line with previous observations (Witzel, Racey, & O’Regan, 2017; Wuerger et al., 2015), there was a small shift toward blue (triangle belonging to the grey disk in the centre of Figure 3(a)).

For adjustments with chromatic illuminations, there were undershoots (i.e., adjustments with lower chroma than the illumination) for all hue directions except for the blue direction (i.e., in the third quadrant in Figure S3(b)). As a result, average adjustments were less saturated. Only average adjustments for illuminations with a bluish hue coincided almost exactly with the illumination colour.

Another feature of the average adjustments (black triangles in Figure 3(a)) was that they are all shifted toward blue. There is no such effect in the yellow direction of the daylight locus. To capture this overall shift toward blue, we identified the blue with the smallest shift, where the average adjustment was almost exactly the same as the illumination colour. This was the case for the blue colour located directly under the daylight locus in Figure 3(a), henceforth *daylight blue*.

To quantify the shift of adjustments toward daylight blue, we calculated the distance of each illumination colour and each adjustment to daylight blue. Then, we determined the blue shift as the difference between the distance of an adjustment and of the corresponding illumination colour from daylight blue. A positive blue shift means that the adjustment was closer to daylight blue than the illumination colour. These blue shifts are illustrated by Figure 3(b). Almost all blue shifts were above zero, indicating a shift toward daylight blue. A t-test across colours indicated that blue shifts were significantly above zero, *t*(40) = 9.5, *p* < .001. For further specification of the blue shift, Figure S4 illustrates the rotations of average adjustments toward daylight blue. The further the illumination colour was away from daylight blue, the more the adjustment was shifted toward daylight blue. This is shown by a highly significant correlation between the blue shift and the distance of the illumination colour from daylight blue, *r*(39) = − 0.82, *p* < .001.

#### Colour constancy

**Figure 4.**
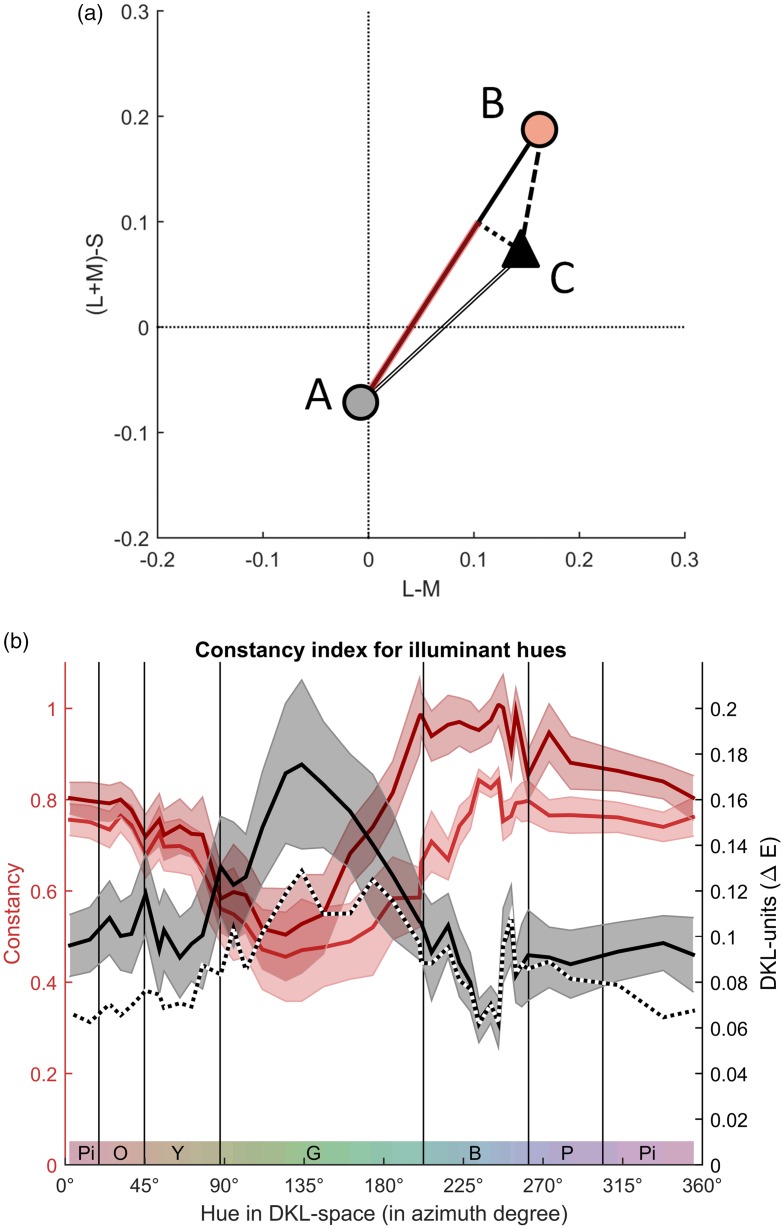
Colour constancy. (a) Illustration of the calculation of colour constancy measures. The grey disk A depicts the neutral illumination colour, the orange disk corresponds to the respective chromatic illuminant colour B, and the black triangle to the achromatic match C. The colour constancy index (CCI) is the distance BC divided by AB. The Brunswick ratio (BR) consists of the projection from AC to AB (red line) divided by AB. (b) The colour constancy measures obtained in our study (red y-axis on the left) as a function of azimuth (x-axis). The dark red curve shows the Brunswick ratio and the light red curve shows the Colour Constancy Index. The black curve and the black-and-white dotted curve correspond to the adjustment error (i.e., BC in (a)) and to interobserver variation (see text for explanation). The corresponding black y-axis on the right represents Euclidean distances in DKL-space. Note that the data along the solid black curve correspond to the length of the black lines in Figure 3(a) and form the basis of the CCI (red curve).

Colour constancy is perfect when achromatic matches (black triangles in Figure 3(a)) coincide with the colour of the achromatic reflectance under the respective chromatic illumination (coloured disks in Figure 3(a)), and lower the further away the adjustments are from the colour of the achromatic reflectance (length of black lines in Figure 3(a)). We consider this distance as a raw measure of colour constancy, or rather of colour ‘inconstancy’, and will refer to it as the *adjustment error*. The adjustment error is plotted as a function of azimuth in Figure 4(b; black curve).

Based on the adjustment error, we calculated two more specific measures of colour constancy, the *Colour Constancy Index* (Arend, Reeves, Schirillo, & Goldstein, 1991) and an adaptation of the *Brunswick ratio* (Olkkonen, Hansen, & Gegenfurtner, 2008; Troost & de Weert, 1991). The calculation of these measures is illustrated by Figure 4(a; see also Foster, 2011). The Colour Constancy Index (CCI) is based on the ‘relative adjustment error’. To calculate the Colour Constancy Index, the adjustment error (black curve in Figure 4(b)) is divided by the illumination shift, that is, the distance between the achromatic reflectance (Neutral 6.5) under a chromatic illumination (coloured disk in Figure 3(a)) and under the neutral illumination (grey disk in Figure 3(a)). This ratio is one if the adjustment error is as large as the illumination shift. This indicates a complete absence of colour constancy. To obtain an index that reflects colour constancy, this value is subtracted from one, so that one corresponds to complete colour constancy:

(1) $$\mathrm{CCI}=1-\frac{|C-B|}{|A-B|}$$

where *A*, *B*, and *C* correspond to the points in Figure 4(a).

The Brunswick ratio assumes that the only systematic variation in colour constancy occurs along the direction of the illumination shift and all other variation of adjustments is due to noise. The adjustments are projected on the direction of the illumination shift (i.e., the direction from the grey disk to the respective coloured disk in Figure 3(a)), resulting in the distance AC′ according to the nomenclature of Figure 4(a). As for the Colour Constancy Index, this distance is expressed relative to the illumination shift (AB):

(2) $$\mathrm{BR}=\frac{|A-C'|}{|A-B|}$$

The advantage of the Colour Constancy index is that it does not need to assume that all deviations of adjustments from the target colour defined by the illumination shift are noise. Another advantage is that, while the Colour Constancy Index is sensitive to precision, the Brunswick ratio is exclusively based on accuracy. The disadvantage of the Colour Constancy Index compared with the Brunswick ratio is that it does not disentangle systematic biases in adjustments and noise, implying that it cannot reach a value of 1 (perfect constancy) in empirical measurements which necessarily involve measurement noise. These conceptual differences are visible in Figure 4(b). The Brunswick ratio is higher than the Colour Constancy Index in bluish regions of colour space, in which there is overshoot in the adjustments (third and fourth quadrant in Figure S3(b)).

Finally, following Witzel et al. (2016), we determined the interindividual variation of adjustments as an index of the precision independent of the congruence with a target colour (black-white dotted curve in Figure 4(b)). The interindividual variation is calculated as the mean differences of each individual observer’s adjustment from the average across observers (similar but not the same as the standard deviation, which is the grey shade in Figure 4(b)). This measure is particularly interesting when examining the relationship between achromatic adjustments and uncertainty.

Despite the conceptual differences between the four measures, all curves indicate that colour constancy is comparatively high in the blue region of colour space and maximal for daylight blue (see red arrow in Figure 3(a) and highest peak of colour constancy close to the blue-purple boundary in Figure 4(b)). This is due to the fact that the adjustment error (black curve in Figure 4(b)) and the interindividual variation (dotted curve and grey shade in Figure 4(b)) are minimal for daylight blue. Hence, adjustments are not only closer to the illumination colour but there is also less uncertainty about the appearance of the achromatic colour when the illumination is daylight blue.

#### Comparison with illumination discrimination and chromatic detection

We then tested the idea that observers discounted for the illuminant to accomplish the achromatic adjustments. For this purpose, we compared colour constancy of achromatic adjustments in this study with the illumination discrimination thresholds measured in the companion study for 12 of the 40 illumination colours (Weiss, Witzel, et al., under review). In case illumination estimation matters for achromatic adjustments, colour constancy should be higher for illuminations that are easy to perceive (low illumination discrimination thresholds). Hence, illumination discrimination thresholds measured in the companion study should be negatively correlated with the Colour Constancy Index and the Brunswick ratio, and positively correlated with the adjustment error and the interobserver variation across the 12 illumination colours (see Table S2 for details). The correlation between Brunswick Ratio and illumination discrimination thresholds was close to significance, *r*(10) = 0.53, *p* = .08, but was positive and hence contradicted the hypothesis. None of the other measures were correlated with illumination discrimination thresholds (all *p* > .71).

In general, any adjustment depends on the ability to perceive colour differences, and hence on discrimination thresholds. In particular, the standard deviations of adjustments may be translated into just-noticeable differences. In achromatic adjustments, these differences are presumably differences to the adapting white-point and mainly concern detection thresholds. To test for a relationship between achromatic adjustments and colour detection, we calculated correlations between the detection thresholds measured in the companion study and the above four measures of colour constancy. However, there was no significant correlation (all *p* > .26), indicating that achromatic adjustments are not related to detection thresholds in a simple way.

**Figure 5.**
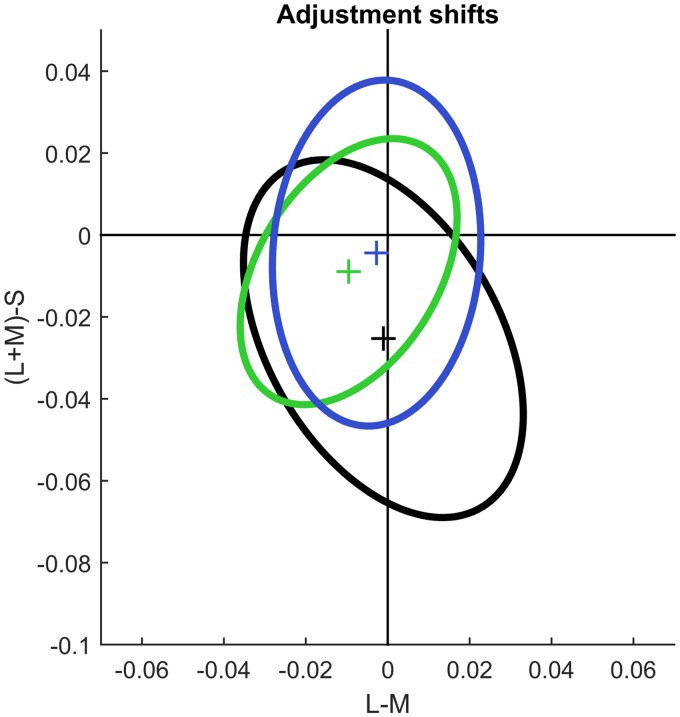
Comparison with chromatic detection and discrimination thresholds. Ellipses are fitted to the adjustment shifts (black ellipse), illumination discrimination thresholds (green), and chromatic detection thresholds (blue). Note the strong blue shift for achromatic adjustments.

In the companion article, we reported a blue shift for illumination discrimination that could partly be explained by a blue bias in chromatic detection (Weiss, Witzel, et al., under review). To compare the overall blue bias in achromatic adjustments to the bias obtained for illumination discrimination and detection thresholds, we integrated all shifts of achromatic adjustments away from the illumination colour. To do so, we subtracted the respective illumination colour (coloured disks in Figure 3(a)) from the respective achromatic adjustment (black triangles in Figure 3(a)). In this way, the deviations between achromatic adjustments and illumination colours (black lines in Figure 3(a)) are relative to the origin. We will call these measures *adjustment shifts*. We fitted an ellipse to the adjustment shifts to capture their overall tendencies (black ellipse in Figure 5) and compared the centre and orientation of the ellipse to the centres and orientations of the ellipses fitted to illumination discrimination (green ellipse) and detection thresholds (blue ellipse) from the companion study.

**Figure 6.**
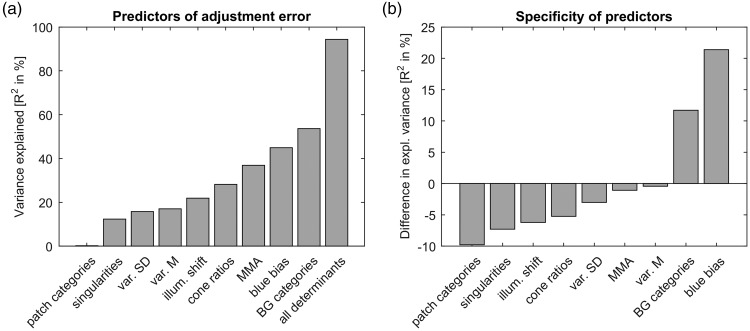
Contribution of each determinant. (a) Variance of adjustment errors explained by each determinant. The x-axis lists different determinants ordered by the variance they explain. The y-axis represents the variance in percent explained by these determinants. All determinants and the adjustment error were computed in CIELAB. The first seven bars correspond to the correlations of the adjustment errors with naming consistencies for the induced colour on the patch (categories: patch), sensory singularities (singularities), average and standard deviation of colour variegation of distractors (var. M and var. SD), metamer mismatch areas (MMA), cone ratios, illumination shift (illum. shift), the distance of the illumination hue to daylight blue (blue bias), and the naming consistency for illumination in the background of the stimulus display (categories: BG). The right-most bar illustrates the variance explained by a multiple regression with all determinants together as predictors. (b) Difference between average explained variance of adjustment errors of all multiple regressions with and without each predictor. The difference of average R2 in percent is shown along the y-axis. Otherwise, the format is the same as in Panel a.

As for chromatic detection thresholds (blue cross in Figure 5), the centre of the adjustment shifts (black cross in Figure 5) is shifted toward the S-pole of the (L + M)-S axis, but the shift is much larger for achromatic adjustments than for detection thresholds. However, while the ellipse for detection thresholds is aligned with the (L + M)-S axis, the orientation of the ellipse for adjustment shifts is oblique to the axes. Other than the blue shift, there does not seem to be any other commonality between achromatic adjustments and the other two measures.

#### Candidate determinants

We then investigated several other determinants that potentially explain the variation of achromatic adjustments and colour constancy. We focused on the Colour Constancy Index as a measure of colour constancy because we observed systematic shifts of adjustments toward the blue direction, and the Brunswick ratio is insensitive to these shifts due to the projection on the illumination shift. To assess the variation of adjustments perceptually, we recalculated adjustments errors (black curve in Figure 4(b)), interindividual variation of adjustments (black-dotted curve in Figure 4(b)), and the Colour Constancy Index (light red curve in Figure 4(b)) in CIELAB space. We assumed that the observer’s adapting white point was the illumination of each scene and used the respective chromatic illuminations as white points for the CIELAB calculations. Because of the strong variation of the white point, we did not use CIELUV because it provides an unreliable chromatic adaptation and hence Euclidean differences strongly change across colour space for different white points. The colour constancy index is largely the same in DKL and CIELAB colour space (Figure S5) and hence allows tests independent of colour spaces.

As candidate determinants, we examined illumination shifts, blue bias, colour categories, metamer mismatch areas, sensory singularities, and cone ratios. As an overview, Figure 6(a) illustrates the variance of the adjustment errors explained by each determinant.

##### Illumination shifts

As can be seen from Figure 3(a), the illumination shift is not the same for all hue directions (this is also true in CIELAB; see Figure S5). The larger the shift, the more colour constancy must be performed by the observer. Hence, failures of colour constancy might be expected to be higher for larger shifts. This was indeed the case (Table S3): illumination shifts were correlated with adjustment errors, *r*(38) = .47, *p* = .002, cf. ‘illum. Shift’ in Figure 6(a), and interindividual variation in CIELAB, *r*(38) = .43, *p* = .005.

##### Blue bias

Above we observed a blue bias, according to which adjustments were shifted toward blue in DKL-space (see Figure 3(b)). To assess the importance of the blue bias for our measures of colour constancy, we determined the distance between the blue daylight illumination (see arrow in Figure 3(a)) and the colour of each other illumination (see coloured disks in Figure 3(a)) in CIELAB. We then calculated correlations between those distances from daylight blue and our three measures of colour constancy (Table S3). The distance from blue daylight was negatively correlated with the CCI, *r*(39) = − .52, *p* < .001, and positively with the adjustment error, *r*(39) = .67, *p* < .001, (cf. ‘blue bias’ in Figure 6(a)), and the interindividual variation, *r*(39) = .57, *p* < .001. These results indicate that constancy decreases with distance to the blue daylight illumination.

##### Colour categories

Figure 4(b) shows that colour constancy changes rather smoothly across hues. This contradicts the idea of category effects on colour constancy, which would imply abrupt changes at category borders and/or at category prototypes. Further analyses also showed that colour constancy did not systematically differ between colours at the boundaries and colours in the centre of the categories. This is further illustrated by Figure S7 in the Supplementary Material.

However, the boundaries shown in Figures 2 and 4(b) are merely hue boundaries. Since colours are not very saturated, category membership is not always maximal at the centres of those hue boundaries (see observer consistency and response times in Figure 2(a) to (b)). According to the idea of categorical colour constancy, category membership and colour constancy should be positively related (see also Witzel et al., 2016). We used category consensus as a measure of category consistency (Figure 2(a) and (b)) and correlated it with each of our three measures of colour constancy. Since response times provided an alternative measure of category membership, we also calculated correlations for response times. We calculated these correlations for both, category membership of induced patch colours and of inducing background colours (see Table S3 for details). Note that the colour names for the induced colours of the patches are shifted by approximately 180° compared with the background naming (Figure 2(b)).

Consensus for naming the patch colours was not correlated to any of the three measures (all *p* > .15; cf. ‘Patch categories’ in Figure 6(a)), but response times were correlated to the interindividual differences, *r*(38) = .37, *p* = .02. In contrast, category consensus for naming background colours was significantly negatively correlated with the colour constancy index, *r*(38) = − .61, *p* < .001, and positively with the adjustment error, *r*(38) = .73, *p* < .001 (cf. ‘BG categories’ in Figure 6(a)), and interindividual variation, *r*(38) = .70, *p* < .001. Response times were also significantly correlated with all of these three measures (all *p* < .007; cf. Table S3). These correlations imply that colour constancy is lower for illumination hues with high naming consensus. This result contradicts the idea of categorical colour constancy, which predicts the inverse.

##### Metamer mismatching

We calculated metamer mismatch volumes in CIELAB for the light achromatic reflectance (Neutral 6.5) that reflects the illumination colour (disks in Figure 3(a)) for the 40 changes from neutral to each of the chromatic illuminations. The calculation of metamer mismatch volumes has been described previously (Logvinenko et al., 2014; Logvinenko et al., 2015; Witzel et al., 2016). We focus on the two-dimensional projections of the volumes on the chromatic plane (i.e., metamer mismatch areas) because observers could not adjust lightness (Witzel et al., 2016). However, results were similar with the three dimensional volumes.

If the uncertainty represented by the metamer mismatch areas were related with the uncertainty of achromatic adjustments, there should be a positive correlation with our measures of adjustment errors and interindividual variation and a negative correlation with the colour constancy index. Table S3 provides details on correlations. There was no significant correlation between metamer mismatch areas and the colour constancy index, *r*(38) = − .13, *p* > .42, but we found a positive correlation with the adjustment error, *r*(38) = .61, *p* < .001, as well as with interindividual variation, *r*(38) = .58, *p* < .001. The absence of a relation with the colour constancy index indicates that metamer mismatch areas are related to the illumination shift, which is taken into account when calculating the colour constancy index. We found a high correlation between metamer mismatch areas and the illumination shift, *r*(38) = .92, *p* < .001. When controlling for illumination shift in a partial correlation, metamer mismatch areas were significantly correlated with all three measures of colour constancy (all *p* < .003; see Table S3 for details). The results support the idea that larger metamer mismatch areas lead to lower colour constancy, as suggested previously (Witzel et al., 2016).

##### Sensory singularities

We determined sensory singularities for the Munsell-like reflectances that cancel the chromatic illumination so as to produce colorimetric grey. For the calculations, we used the programs provided by Witzel et al. (2015). The idea is that adjustments should be more accurate and precise if these reflectances are singular because singularity makes the colour signal of these reflectances more predictable. However, correlations between sensory singularities and measures of colour constancy did not support this idea (see Table S3). Sensory singularities were not correlated to the CCI (*p* > .35), but singularities were positively correlated with the adjustment error, *r*(38) = .35, *p* = .026, and interindividual variation, *r*(38) = .41, *p* = .008. Positive correlations contradict the idea that sensory singularities reduce adjustment errors and variation. The observed correlations may be explained by the role of chroma since sensory singularities are strongly related to differences in chroma (Witzel et al., 2015). When controlling for chroma (which is largely equivalent to the illumination shift in the present study), none of the measures was correlated with sensory singularities anymore, all *r*(38) < .22, *p* > .17.

##### Cone ratios

We also calculated the cone ratios for the 39 distractor and 2 background colours for each change from the neutral to each of the 40 illuminations. First of all, we observed that cone excitations for all three types of cones and all 40 illumination changes were almost perfectly correlated, min. *r*(39) = .88, max. *r*(39) = 1, implying that cone ratios are almost constant (cf. Figure 2 in Foster & Nascimento, 1994).

Following the approach of Nascimento et al. (2004), we calculated predictions of the adjusted colour under the respective chromatic illuminations based on the 41 cone ratios of the surrounding colours. If colour constancy was determined by cone ratios, observers’ adjustments should be closer to the predictions based on cone ratios than to the actual colour of the surface under each chromatic illumination. We determined the distance between the cone ratio prediction (averaged across the 41 estimations) and the average achromatic adjustment for each illumination, and compared them with the adjustment error across the 40 illuminations in a paired *t*-test (analoguous to Figure 10 in Witzel et al., 2016). Results showed that adjustments were further away from the cone ratio predictions than from the predictions based on the light reflected of the grey reflectance (Neutral 6.5) under the chromatic illuminations, *t*(39) = 25.2, *p* < .001.

Then, we determined the distance between the 41 predictions and the actual illumination colour (on the test patch) according to the Munsell-like reflectances and the Gaussian illuminations. The average of these distances provides the prediction error of the cone ratio predictions for each illumination. We calculated the correlations between cone ratio prediction error and the Colour Constancy Index, the adjustments error, and interindividual variability (see Table S3). The prediction error was correlated with the adjustment error, *r*(38) = .53, *p* < .001, and with the interindividual variation, *r*(38) > .53, *p* < .001, but not with the Colour Constancy Index, *r*(38) = − .11, *p* = .48.

These results suggest that the more the illumination colour deviates from the cone ratio prediction, the more adjustments deviate from the illumination colour and the more variable adjustments are across individual observers. Since the Colour Constancy Index accounts for the illumination shift, the absence of a correlation between cone ratio predictions and the Colour Constancy Index indicates that the correlations with the raw distance measures (adjustment error and interindividual variation) are due to the variation of illumination shifts (cf. Figure S6). In fact, cone ratio prediction errors were correlated to the size of illumination shifts, *r*(38) = .80, *p* < .001. However, when controlling for illumination shifts in partial correlations, cone ratio prediction errors were still positively correlated with individual variation, *r*(38) = .35, *p* = .03), but not with the Colour Constancy Index and adjustment errors (both *p* > .07; see Table S3).

##### Colour variegation

To test for the effect of colour variegation on colour constancy, we calculated how strongly the distractor colours differed from the background colours. For this, we calculated the chroma of all distractor colours in each stimulus display, assuming the illumination colour as the white point. The average chroma indicates the overall contrast of distractors to the background, and the standard deviation corresponds to the variation in contrast. These two factors were uncorrelated, *r*(38) = 0.06, *p* = .70. The average chroma of the distractors (var. M in Figure 6(a)) was positively correlated with the colour constancy index, *r*(38) = 0.37, *p* = .02, and negatively with the adjustment error, *r*(38) = − 0.41, *p* = .008, and individual variation, *r*(38) = − 0.50, *p* = .001. The standard deviation was negatively correlated with the adjustment error, *r*(38) = − 0.40, *p* = .01, but was not correlated with the Colour Constancy Index, *r*(38) = 0.21, *p* = .19, and individual variation, *r*(38) = − 0.29, *p* = .07. The above correlations suggest that colour constancy tends to be higher, the more distractor colours contrast with the background.

##### Combination of determinants

To assess the variance explained by the combination of all determinants, we calculated a multiple regression (last bar in Figure 6(a)). All determinants together explained *R*^2 ^= 94.4% of the variance of the adjustment error, *F*(9, 30) = 56.0, *p* < .001. The two most important determinants were the distance from daylight blue (blue bias: *R*^2 ^= 44.9%) and the consistency in naming the illumination colour (BG categories: *R*^2 ^= 53.7%). These two determinants were correlated with each other, *r*(38) = .44, *p* = .005, but each of them were still correlated with the adjustment error when controlling for the respective other determinant; BG categories: *r*(38) = .65, *p* < .001, distance from daylight blue: *r*(38) = .57, *p* < .001. These two factors together explained *R*^2 ^= 68.7% of the variance of adjustment errors, *F*(2, 32) = 40.7, *p* < .001. Eliminating the distance from daylight blue from the multiple regression with all determinants reduced the explained variance from 94% to 71%. This shows that this factor is not redundant in the regression model. In contrast, eliminating the consistency in illumination naming (BG categories) barely reduced the explained variance (0.08% of 94%), implying that this factor is fully accounted for by the combination of all the other factors.

To account for the complex interrelationships between predictors, we compared the contribution of each predictor to regression models without the respective predictor. For this, we calculated multiple regressions for all possible combinations of predictors. For each predictor, we found differences between the average explained variance of all models with that predictor and the average of all the other models without that predictor. Figure 6(b) illustrates these differences. Differences above zero show that the respective predictor makes a contribution to the explained variance that cannot be accounted for by the combination of other factors. Only the blue bias and illumination naming yield positive differences, confirming that these two factors play a major role in explaining the variance of adjustment errors.

## Discussion

### Daylight and Blue Bias

Our results showed a systematic shift of achromatic adjustments toward the blue direction of the daylight locus (Figure 3(b) and Figure 5). The closer the colour of an illumination was to daylight blue, the closer adjustments were to the colour of the grey reflectance under the other illumination. Adjustments also tended toward undershooting (i.e., shifts toward the colorimetric grey) when colours were away from daylight blue (Figure 3(a)). The distance of each illumination from daylight blue was one of the two most important determinants of adjustment errors, explaining a large proportion of its variance (44.9%).

In general, undershooting may be explained by incomplete adaptation. Adaptation in this setup was only controlled through the patterned background in the stimulus display. This may be too little to guarantee full adaptation as it is obtained by adapting to the illumination colour across the full visual field (see e.g., Hansen et al., 2007). For this reason, undershooting itself is not surprising.

What is particular is that the undershoot disappears almost completely under the blue illumination. Due to the way we designed the stimuli for this experiments, illumination shifts are not completely equal for every hue direction (Figure 3(a)). Although the 40 reflectances yielded the same saturation under the neutral illuminant in DKL-space, the chromatic illuminations needed slightly different levels of saturation to cancel the saturation of those reflectances and produce a colorimetric grey under every illumination. In DKL-space (Figure 3(a)), the daylight blue illuminant yielded one of the largest illumination shifts (difference between grey and coloured disk), and illumination shifts were negatively correlated with the blue bias in Figure 3(b), *r*(38) = − .60, *p* < .001. This implies that higher blue shifts appear with smaller illumination shifts. The contrary would be expected if illumination shifts increased the blue bias by furthering undershoots. To double check, we also inspected illumination shifts in CIELAB (Figure S6) and found that illumination shifts seem to be small for blue illuminations; at the same time, they are also small for yellow illuminations and yet there is only a shift toward blue, but not toward yellow. Consequently, the blue bias cannot be explained by illumination shifts.

We discussed for our colour naming experiment above, that blue illuminations might be less saturated and weaker inducers. This idea is at least partially in line with previous observations with simple chromatic contrast displays according to which colour induction was weaker when inducing backgrounds had colours along the daylight axes in DKL-space (Klauke & Wachtler, 2015). However, the blue bias observed here contradicts this idea. If induction was weakest for blue illuminations, adjustments should be less shifted away from colorimetric grey. Instead, observers’ adjustments were most strongly shifted, resulting in almost perfect coincidence of adjustments with the illumination shift and highest colour constancy under blue illuminations. For this reason, the blue bias may not be explained by the anisotropy of colour space or weaker induction by bluish illuminations.

As summarised in the Introduction section, previous studies found inconsistent results concerning the variation of colour constancy across illumination hues (Brainard, 1998; Daugirdiene et al., 2016; de Almeida et al., 2004; Delahunt & Brainard, 2004; Hansen et al., 2007; Logvinenko & Tokunaga, 2011; Olkkonen et al., 2009; Olkkonen et al., 2010; Schultz et al., 2006). To control effects of hues, illumination, and reflectance spectra, we used a large range of 40 illumination hues, smooth illumination spectra to avoid unpredictable effects of spectral discontinuities and carefully controlled surface colour shifts. It is still possible that results are affected by the fact that we used Munsell-like reflectances and artificial Gaussian illuminant spectra rather than naturally occurring surface and illumination spectra.

However, it is difficult to control parameters of natural spectra across colour space, in particular since certain spectra, such as turquoise illuminants, barely exist in the natural environment. More importantly, natural reflectances and illuminants typically have rather smooth spectra and should be well approximated by our technique. The blue bias was not particular to single illumination directions, but occurred across the ensemble of 40 illuminant spectra. Even if there were single spectra in our set of illuminants that might have unnatural spectral properties, they could not explain the observed blue bias. For these reasons, we expect that the blue bias for achromatic adjustments in this experiment is likely to occur in the natural environment. Hence, our observation that colour constancy is highest for daylight blue illuminations suggests that colour constancy is optimised for such blue daylight illuminations.

The question arises of where this blue bias comes from. One possibility is that it is built into the visual system, and in particular is a feature of adaptation to different hue directions. However, the fact that the bias is toward daylight blue rather than the S-pole of the second-stage mechanisms suggests that the effect is not due to asymmetric adaptation along the (L + M)-S axis (Delahunt & Brainard, 2004; Foster, Amano, & Nascimento, 2003). Another possibility is that observers have more experience with illumination shifts along the daylight axis (Pearce et al., 2014), but this is inconsistent with our results that show the effect does not occur in the yellow direction of the daylight axis.

### Perceived Illumination and Colour Constancy

Our results revealed clear differences between achromatic adjustments and perceived illumination as measured through illumination discrimination thresholds (Figure 5). Apart from the blue bias, we did not find any relationship between achromatic adjustments and illumination discrimination thresholds. This suggests that the ability to discriminate illuminations is of minor importance for colour constancy, at least when measured by achromatic adjustments.

The observation that colour constancy is unrelated to perceived illumination is in line with previous studies according to which observers are surprisingly bad in estimating the illumination (de Almeida & Nascimento, 2009; Granzier, Brenner, & Smeets, 2009). Taken together, these findings put into question the idea that observers consciously discount the illuminant.

At the same time, recent studies (Aston et al., 2016; Pearce et al., 2014; Radonjić et al., 2016) suggest that illumination discrimination may be considered as a measure for colour constancy because implicit mechanisms compensate for the effects of the illuminant change. In the companion study (Weiss, Witzel, et al., under review), we observed a strong correlation between chromatic detection and illumination discrimination, *r*(10) = .72, *p* = .009, indicating that illumination discrimination may be explained to a large degree by asymmetries in chromatic sensitivity.

In contrast, the present study showed that colour constancy (as measured through achromatic adjustments) is neither related to illumination discrimination nor to chromatic sensitivity. This was the case even though the present measurements included exactly the same stimulus displays as the companion study (Weiss, Witzel, et al., under review). Since achromatic adjustments measure colour constancy, the observation that they are unrelated to illumination discrimination casts doubt on the idea that illumination discrimination directly translates into colour constancy. In contrast to illumination discrimination, achromatic adjustments cannot be explained by chromatic sensitivity. This suggests that the large blue bias observed for achromatic adjustments might be qualitatively different from the blue bias in the chromatic sensitivity and illumination discrimination.

If this is so, our findings also inform us about the idea that colour appearance is uncertain along the daylight locus (Beer et al., 2006; Bosten et al., 2015; Gegenfurtner et al., 2015; Lafer-Sousa et al., 2015; Winkler et al., 2015; Witzel et al., 2011). Previous experiments found that achromatic adjustments under neutral illumination and adaptation vary most strongly along the daylight axis (Beer et al., 2006; Bosten et al., 2015; Witzel et al., 2011) and are shifted on average toward blue (Winkler et al., 2015; Wuerger et al., 2015). Our experiments extend these observations by showing that this shift toward blue is stronger the further the illumination colour is away from blue and it disappears when the illumination is blue. This asymmetry speaks against a general effect along the daylight axis. Our observation that achromatic adjustments are not related to illumination discrimination suggests that this blue bias is not due to uncertainty about the illumination.

According to Winkler et al. (2015), the asymmetry along the blue-yellow direction is due to observers’ tendency to attribute bluish colours to the illumination and yellowish colours to objects and surfaces. This is in line with the observation that colour constancy in our achromatic adjustments is highest for blue, because this shows that observers completely attribute the blue colour to the illumination. High colour constancy for daylight blue can be particularly helpful to recognise objects under shadow because shadows shed blue lights on objects (Churma, 1994; Troscianko et al., 2009). Hence, the blue bias could be an adaptation to the natural environment.

### Categorical Colour Constancy

Our findings contradicted the patterns of categorical colour constancy. First of all, our results contest the idea that adjusted colours are shifted toward prototypes in colour constancy, as they do in colour memory (Bae et al., 2015). In our experiment, achromatic adjustments were not shifted toward category centres. Instead, all adjustments were shifted toward blue (see Results section Patterns of Adjustments).

Our findings also undermined the idea that colour constancy is highest around category centres and prototypes and decreases toward the boundaries of colour categories, as has been suggested by a study using asymmetric matching (Kulikowski & Vaitkevicius, 1997) and by studies on category constancy (Olkkonen et al., 2009; Olkkonen et al., 2010). In contrast to those studies, a previous study, using asymmetric matching, did not find evidence for higher constancy within the categories or any other relationship between colour constancy and colour categories (Witzel et al., 2016). Using achromatic matching, the present study found a negative relationship between colour constancy and category membership, which completely contradicts categorical colour constancy.

These diverging results across studies indicate that the relationship between colour constancy performance and colour categories depends on the methods and setups used to measure colour constancy, rather than being a general feature of colour constancy. In particular, the results of our study can be explained by different degrees of adaptation depending on the illumination hue. If observers adapt most easily to the blue daylight illumination, their colour constancy is high. At the same time, the colours of this illumination look more desaturated due to adaptation and hence are named less consistently. The inverse is true for illumination colours far off daylight blue, if observers only achieve lower levels of adaptation for those illumination hues.

The idea that both the blue bias and category consistency are due to a common source, namely different levels of adaptation, is supported by the fact that both measures are correlated. At the same time, each of these two determinants contributes to the explanation of achromatic adjustments and colour constancy, when controlling for the respective other determinant. These results make sense if we consider that the blue bias and the category consensus capture different aspects of the variation of adaptation across illumination hues. If this is true, future experiments that specifically measure the variation of adaptation across illumination hues might reveal the origin of the patterns we observed for colour constancy with respect to the blue bias and category consensus.

### Other Determinants

Other factors besides the blue bias and category consistency, such as metamer mismatch areas, cone ratios, and colour variegation, yielded correlations with some or all of our measures of colour constancy (see also Figure 6). The correlation of metamer mismatch areas with adjustment errors and interindividual variation confirms the results found with asymmetric matches (Witzel et al., 2016). This observation is in line with the idea that higher uncertainty due to metamer mismatching leads to higher variability of adjustments, implying higher deviations from a target colour and higher variation across observers.

The results for cone ratios are in line with previous findings (Foster & Nascimento, 1994; Foster et al., 1997; Nascimento et al., 2004). Our observations suggest that colour constancy is worse for displays with higher cone ratio prediction errors, suggesting that human observers might use cone ratios to achieve colour constancy (Nascimento et al., 2004).

The role of colour variegation in our measures of colour constancy is in line with the observation that variegation of surrounding colours modulates the appearance of target colours (Brown & MacLeod, 1997). The observed correlations imply that colour constancy increases with higher levels of variegation (Golz, 2010; Linnell & Foster, 2002). This result may be explained by the idea that higher levels of colour variegation are more informative about the test colour because there are more cues about its relation to other colours.

The evaluation of the precise contribution of the factors beyond the blue bias and illumination naming is complicated by their complex interaction. To disentangle their role in colour constancy, it would be good to conduct experiments that specifically test each of these factors while controlling for the others.

Another potential limitation of our study is that colour constancy in real instead of simulated scenes may involve factors beyond the ones we investigated here for achromatic adjustments. For example, memory colours might play a role (Granzier & Gegenfurtner, 2012). Real scenes may lead observers to make inferences to estimate surface colours, which might not play a role for the induction effects in our simplified displays. In particular, illumination change is self-evident to the observer in real scenes while this is not necessarily true for rendered scenes. Hence, effects that compensate for simulated illumination changes might focus on particular aspects of colour constancy, such as adaptation and local contrast, to the detriment of other factors that might be particular to real illumination change. In fact, while we observed systematic undershoots in most hue directions, colour constancy with real scenes can be almost perfect (Allred & Olkkonen, 2013; Olkkonen et al., 2009; Olkkonen et al., 2010; Weiss, Bloj, & Gegenfurtner, under review), indicating that other factors might further increase colour constancy.

At the same time, through our simplified displays, we identified factors that modulate colour appearance depending on the chromatic context and are most likely to play a similar role in real scenes where the context changes in a comparable way. Rendering the colours of simulated scenes made it possible to collect fine-grained measurements of colour appearance for a large number of hue directions and to control important characteristics of reflectance and illumination spectra. Additional factors might contribute to colour constancy in real scenes. Nevertheless, the blue bias and differences in adaptation as captured by naming consistency modulate colour appearance in a way that cannot be explained away by the realism of the stimulus displays. It would be interesting to test the role of the determinants identified here in more realistic settings.

## Conclusion

Using achromatic adjustments, we investigated colour constancy for chromatic illuminations along 40 different colour directions. We also measured colour categories for the induced colour of the test patch and of the illumination colour.

Although we found some small but systematic differences between the different colour categories, the results of the naming experiment generally confirm the idea that colours induced by colour contrast are shifted to the opponent hue direction predicted by the second-stage mechanisms. Results also provided some evidence that bluish illuminations are seen as less saturated, which is in line with the blue bias for illumination estimation observed previously (Aston et al., 2016; Pearce et al., 2014; Radonjić et al., 2016; Weiss, Witzel, et al., under review).

In the achromatic adjustment experiment, we observed a strong blue bias: Independent of the illumination colour, adjustments were strongly shifted toward the blue pole of the daylight axis. Average colour constancy was perfect under the blue daylight illumination, but not in the other hue directions, due to undershooting and the blue shift. Our findings support the idea that colour constancy is optimised for bluish, but not for yellowish daylight.

We also observed a negative relationship between colour constancy and consistency of naming the illumination colour. This relationship is in conflict with the idea of categorical colour constancy. Instead, it suggests that observers more easily adapt to bluish illuminations and categorise them as grey.

Colour constancy was not related to illumination discrimination and chromatic detection. There was some evidence that other factors, such as metamer mismatching, relational colour constancy, and colour variegation, play a role in colour constancy, but in a rather complicated way. In any case, the blue bias and the consistency of the illumination categories explained most of the variance of the achromatic adjustments.

## Supplementary Material

Supplementary material

## References

[bibr1-2041669517739635] Allred S. R. Olkkonen M. (2013) The effect of background and illumination on color identification of real, 3D objects. Frontiers in Psychology 4: 821 doi: 10.3389/fpsyg.2013.00821. 24273521 10.3389/fpsyg.2013.00821 PMC3823087

[bibr2-2041669517739635] Arend L. E. Reeves A. Schirillo J. Goldstein R. (1991) Simultaneous color constancy: Paper with diverse Munsell values. Journal of the Optical Society of America A 8: 661–672. 10.1364/josaa.8.000661 2045968

[bibr3-2041669517739635] Aston S. Turner J. Le Couteur Bisson T. Jordan G. Hurlbert A. (2016) Better colour constancy or worse discrimination? Illumination discrimination in colour anomalous observers. Perception 45 , (issue 2 supplement), 207.

[bibr4-2041669517739635] Bae G. Y. Olkkonen M. Allred S. R. Flombaum J. I. (2015) Why some colors appear more memorable than others: A model combining categories and particulars in color working memory. Journal of Experimental Psychology: General 144: 744–763. doi: 10.1037/xge0000076. 25985259 10.1037/xge0000076

[bibr5-2041669517739635] Beer R. D. Dinca A. MacLeod D. I. A. (2006) Ideal white can be yellowish or bluish, but not reddish or greenish. Journal of Vision 6: 417–417. doi: 10.1167/6.6.417.

[bibr6-2041669517739635] Bosten J. M. Beer R. D. MacLeod D. I. (2015) What is white? Journal of Vision 15: 5 doi: 10.1167/15.16.5. 10.1167/15.16.5 PMC4675320 26641948

[bibr7-2041669517739635] Brainard D. H. (1997) The psychophysics toolbox. Spatial Vision 10: 433–436. doi: 10.1163/156856897X00357. 9176952

[bibr8-2041669517739635] Brainard D. H. (1998) Color constancy in the nearly natural image. 2. Achromatic loci. Journal of the Optical Society of America A 15: 307–325. doi: 10.1364/JOSAA.15.000307. 10.1364/josaa.15.000307 9457790

[bibr9-2041669517739635] Brown R. O. MacLeod D. I. A. (1997) Color appearance depends on the variance of surround colors. Current Biology 7: 844–849. doi: S0960-9822(06)00372-1. 9382808 10.1016/s0960-9822(06)00372-1

[bibr10-2041669517739635] Burns S. A. Cohen J. B. Kuznetsov E. N. (1989) Multiple metamers: Preserving color matches under diverse illuminants. Color Research & Application 14: 16–22. doi: 10.1002/col.5080140106.

[bibr11-2041669517739635] Chauhan T. Perales E. Xiao K. Hird E. Karatzas D. Wuerger S. (2014) The achromatic locus: Effect of navigation direction in color space. Journal of Vision 14: 25 doi: 10.1167/14.1.25. 10.1167/14.1.25 PMC3903293 24464164

[bibr12-2041669517739635] Churma M. E. (1994) Blue shadows: Physical, physiological, and psychological causes. Applied Optics 33: 4719–4722. doi: 10.1364/AO.33.004719. 20935843 10.1364/AO.33.004719

[bibr13-2041669517739635] Cohen J. B. Kappauf W. E. (1982) Metameric color stimuli, fundamental metamers, and Wyszecki’s metameric blacks. American Journal of Psychology 95: 537–564. doi: 10.2307/1422186. 7168455

[bibr14-2041669517739635] Daugirdiene A. Kulikowski J. J. Murray I. J. Kelly J. M. F. (2016) Test illuminant location with respect to the Planckian locus affects chromaticity shifts of real Munsell chips. Journal of the Optical Society of America A 33: A77–A84. doi: 10.1364/JOSAA.33.000A77. 10.1364/JOSAA.33.000A77 26974944

[bibr15-2041669517739635] de Almeida V. M. Fiadeiro P. T. Nascimento S. M. (2004) Color constancy by asymmetric color matching with real objects in three-dimensional scenes. Visual Neuroscience 21: 341–345. doi: 10.1017/S0952523804213074. 15518211 10.1017/s0952523804213074

[bibr16-2041669517739635] de Almeida V. M. Nascimento S. M. (2009) Perception of illuminant colour changes across real scenes. Perception 38: 1109–1117. doi:10.1068/p6277. 19817145 10.1068/p6277

[bibr17-2041669517739635] Delahunt P. B. Brainard D. H. (2004) Does human color constancy incorporate the statistical regularity of natural daylight? Journal of Vision 4: 57–81. doi: 10.1167/4.2.1. 15005648 10.1167/4.2.1

[bibr18-2041669517739635] Foster D. H. (2011) Color constancy. Vision Research 51: 674–700. doi: 10.1016/j.visres.2010.09.006. 20849875 10.1016/j.visres.2010.09.006

[bibr19-2041669517739635] Foster D. H. Amano K. Nascimento S. M. C. (2003) Tritanopic colour constancy under daylight changes? In: Mollon J. D. Pokorny J. Knoblauch K. (eds) Normal and defective colour vision, Oxford, England: Oxford University Press, pp. 218–224.

[bibr20-2041669517739635] Foster, D. H., & Nascimento, S. M. (1994). Relational colour constancy from invariant cone-excitation ratios. *Proceedings of the Royal Society B: Biological Sciences, 257*, 115–121. https://doi.org/10.1098/rspb.1994.0103. 10.1098/rspb.1994.0103 7972159

[bibr21-2041669517739635] Foster D. H. Nascimento S. M. C. Craven B. J. Linnell K. J. Cornelissen F. W. Brenner E. (1997) Four issues concerning colour constancy and relational colour constancy. Vision Research 37: 1341–1345. doi: 10.1016/S0042-6989(96)00285-4. 9205725 10.1016/s0042-6989(96)00285-4

[bibr22-2041669517739635] Gegenfurtner K. R. Bloj M. Toscani M. (2015) The many colours of ‘the dress’. Current Biology 25: R543–R544. doi: 10.1016/j.cub.2015.04.043. 25981790 10.1016/j.cub.2015.04.043

[bibr23-2041669517739635] Golz J. (2010) Colour constancy: Influence of viewing behaviour on grey settings. Perception 39: 606–619. doi: 10.1068/p6052. 20677698 10.1068/p6052

[bibr24-2041669517739635] Granzier J. J. M. Brenner E. Smeets J. B. (2009) Can illumination estimates provide the basis for color constancy? Journal of Vision 9: 18.1–18.11. doi: 10.1167/9.3.18;/9/3/18/. 10.1167/9.3.18 19757957

[bibr25-2041669517739635] Granzier J. J. M. Gegenfurtner K. R. (2012) Effects of memory colour on colour constancy for unknown coloured objects. i-Perception 3: 190–215. doi: 10.1068/i0461. 23145282 10.1068/i0461 PMC3485846

[bibr26-2041669517739635] Hansen T. Walter S. Gegenfurtner K. R. (2007) Effects of spatial and temporal context on color categories and color constancy. Journal of Vision 7: 1–15. doi: 10.1167/7.4.2. 10.1167/7.4.2 17461686

[bibr27-2041669517739635] Ishihara S. (2004) Ishihara’s tests for colour deficiency, Tokyo, Japan: Kanehara Trading Inc.

[bibr28-2041669517739635] Kitaoka A. (2017) Strawberries appear to be reddish, though the pixels are not. *Retrieved from*. https://twitter.com/AkiyoshiKitaoka/status/836382313160171521.

[bibr29-2041669517739635] Klauke S. Wachtler T. (2015) “Tilt” in color space: Hue changes induced by chromatic surrounds. Journal of Vision 15: 17 doi: 10.1167/15.13.17. 10.1167/15.13.17 26401624

[bibr30-2041669517739635] Kleiner, M., Brainard, D., & Pelli, D. (2007). What's new in Psychtoolbox-3? *Perception, 36*, (issue 1 supplement), 14. (ECVP Abstract Supplement.).

[bibr31-2041669517739635] Kohonen O. Parkkinen J. Jaaskelainen T. (2006) Databases for spectral color science. Color Research and Application 31: 381–390. doi: 10.1002/Col.20244.

[bibr32-2041669517739635] Kraft, J. M., & Brainard, D. H. (1999). Mechanisms of color constancy under nearly natural viewing. *Proceedings of the National Academy of Sciences USA, 96*, 307–312. doi: 10.1073/pnas.96.1.307. 10.1073/pnas.96.1.307 PMC15135 9874814

[bibr33-2041669517739635] Kulikowski J. J. Vaitkevicius H. (1997) Colour constancy as a function of hue. Acta Psychologica 97: 25–35. doi: 10.1016/S0001-6918(97)00022-X. 9448512 10.1016/s0001-6918(97)00022-x

[bibr34-2041669517739635] Lafer-Sousa R. Hermann K. L. Conway B. R. (2015) Striking individual differences in color perception uncovered by ‘the dress’ photograph. Current Biology 25: R545–R546. doi: 10.1016/j.cub.2015.04.053. 25981795 10.1016/j.cub.2015.04.053 PMC4921196

[bibr35-2041669517739635] Linnell K. J. Foster D. H. (2002) Scene articulation: dependence of illuminant estimates on number of surfaces. Perception 31: 151–159. doi: 10.1068/p03sp. 11922129 10.1068/p03sp PMC1896062

[bibr36-2041669517739635] Livitz G. Riesen G. Shepard T. Mingolla E. Eskew R. (2016) Afterimages and induced colors have the same hue: Implications for discounting illuminants. Journal of Vision 16: 1145 doi: 10.1167/16.12.1145.

[bibr37-2041669517739635] Logvinenko A. D. Funt B. Godau C. (2014) Metamer mismatching. IEEE Transactions on Image Processing 23: 34–43. doi: 10.1109/TIP.2013.2283148. 24108464 10.1109/TIP.2013.2283148

[bibr38-2041669517739635] Logvinenko A. D. Funt B. Mirzaei H. Tokunaga R. (2015) Rethinking colour constancy. PLoS One 10: e0135029 doi: 10.1371/journal.pone.0135029. 26356217 10.1371/journal.pone.0135029 PMC4565710

[bibr39-2041669517739635] Logvinenko A. D. Tokunaga R. (2011) Colour constancy as measured by least dissimilar matching. Seeing and Perceiving 24: 407–452. doi: 10.1163/187847511X588746. 21902878 10.1163/187847511X588746

[bibr40-2041669517739635] Lotto R. B. Purves D. (2002) The empirical basis of color perception. Consciousness and Cognition 11: 609–629. doi: 10.1016/S1053-8100(02)00014-4. 12470626 10.1016/s1053-8100(02)00014-4

[bibr41-2041669517739635] Malkoc G. Kay P. Webster M. A. (2005) Variations in normal color vision. IV. Binary hues and hue scaling. Journal of the Optical Society of America A 22: 2154–2168. doi: 10.1364/JOSAA.22.002154. 10.1364/josaa.22.002154 16277285

[bibr42-2041669517739635] McCamy C. S. Marcus H. Davidson J. G. (1976) A color-rendition chart. Journal of Applied Photographic Engineering 2: 95–99.

[bibr43-2041669517739635] Munsell Color Services (2007) The Munsell Book of Color—Matte collection, Grandville, MI: x-rite.

[bibr44-2041669517739635] Nascimento S. M. de Almeida V. M. Fiadeiro P. T. Foster D. H. (2004) Minimum-variance cone-excitation ratios and the limits of relational color constancy. Visual Neuroscience 21: 337–340. doi: 10.10170S095252380421327X. 15518210 10.1017/s095252380421327x

[bibr45-2041669517739635] Olkkonen M. Hansen T. Gegenfurtner K. R. (2008) Color appearance of familiar objects: Effects of object shape, texture, and illumination changes. Journal of Vision 8: 1–16. doi: 10.1167/8.5.13. 10.1167/8.5.13 18842084

[bibr46-2041669517739635] Olkkonen M. Hansen T. Gegenfurtner K. R. (2009) Categorical color constancy for simulated surfaces. Journal of Vision 9: 1–18. doi: 10.1167/9.12.6. 10.1167/9.12.6 20053097

[bibr47-2041669517739635] Olkkonen M. Witzel C. Hansen T. Gegenfurtner K. R. (2010) Categorical color constancy for real surfaces. Journal of Vision 10: 1–22. doi: 10.1167/10.9.16. 10.1167/10.9.16 21187350

[bibr48-2041669517739635] Parkkinen J. P. S. Hallikainen J. Jaaskelainen T. (1989) Characteristic spectra of Munsell colors. Journal of the Optical Society of America A-Optics Image Science and Vision 6: 318–322. doi: 10.1364/Josaa.6.000318.

[bibr49-2041669517739635] Pearce B. Crichton S. Mackiewicz M. Finlayson G. D. Hurlbert A. (2014) Chromatic illumination discrimination ability reveals that human colour constancy is optimised for blue daylight illuminations. PLoS One 9: e87989 doi: 10.1371/journal.pone.0087989. 24586299 10.1371/journal.pone.0087989 PMC3929610

[bibr50-2041669517739635] Pelli D. G. (1997) The VideoToolbox software for visual psychophysics: Transforming numbers into movies. Spatial Vision 10: 437–442. doi: 10.1163/156856897X00366. 9176953

[bibr51-2041669517739635] Philipona D. L. O’Regan J. K. (2006) Color naming, unique hues, and hue cancellation predicted from singularities in reflection properties. Visual Neuroscience 23: 331–339. doi: 10.1017/S0952523806233182. 16961964 10.1017/S0952523806233182

[bibr52-2041669517739635] Radonjić A. Pearce B. Aston S. Krieger A. Dubin H. Cottaris N. P. Hurlbert A. C. (2016) Illumination discrimination in real and simulated scenes. Journal of Vision 16: 2 doi: 10.1167/16.11.2. 10.1167/16.11.2 PMC5024666 28558392

[bibr53-2041669517739635] Ratnasingam S. Anderson B. L. (2015) The role of chromatic variance in modulating color appearance. Journal of Vision 15: 19 doi: 10.1167/15.5.19. 10.1167/15.5.19 26067537

[bibr54-2041669517739635] Schultz S. Doerschner K. Maloney L. T. (2006) Color constancy and hue scaling. Journal of Vision 6: 1102–1116. doi: 10.1167/6.10.10/6/10/10/. 17132082 10.1167/6.10.10

[bibr55-2041669517739635] Shepard R. N. (1992) The perceptual organization of colors: An adaptation to regularities of the terrestrial world? In: Barkow J. Cosmides L. Tooby J. (eds) Adapted mind, Oxford, England: Oxford University Press, pp. 495–532.

[bibr56-2041669517739635] Smithson H. E. (2005) Sensory, computational and cognitive components of human colour constancy. Philosophical Transactions of the Royal Society of London B: Biological Sciences 360: 1329–1346. doi: 10.1098/rstb.2005.1633. 16147525 10.1098/rstb.2005.1633 PMC1609194

[bibr57-2041669517739635] Speigle J. M. Brainard D. H. (1999) Predicting color from gray: The relationship between achromatic adjustment and asymmetric matching. Journal of the Optical Society of America A 16: 2370–2376. doi: 10.1364/JOSAA.16.002370. 10.1364/josaa.16.002370 10517021

[bibr58-2041669517739635] The MathWorks Inc (2007) *Matlab—The language of Technical Computing (Version**R2007a)*, Natick, MA: Author.

[bibr59-2041669517739635] Troost J. M. de Weert C. M. (1991) Naming versus matching in color constancy. Perception and Psychophysics 50: 591–602. doi: 10.3758/BF03207545. 1780207 10.3758/bf03207545

[bibr60-2041669517739635] Troscianko T. Benton C. P. Lovell P. G. Tolhurst D. J. Pizlo Z. (2009) Camouflage and visual perception. Philosophical Transactions of the Royal Society of London. Series B, Biological sciences 364: 449–461. doi: 10.1098/rstb.2008.0218. 18990671 10.1098/rstb.2008.0218 PMC2674079

[bibr61-2041669517739635] Vazquez-Corral J. O’Regan J. K. Vanrell M. Finlayson G. D. (2012) A new spectrally sharpened sensor basis to predict color naming, unique hues, and hue cancellation. Journal of Vision 12: 7 doi: 10.1167/12.6.7. 10.1167/12.6.7 22665457

[bibr62-2041669517739635] Webster M. A. Miyahara E. Malkoc G. Raker V. E. (2000) Variations in normal color vision. II. Unique hues. Journal of the Optical Society of America A 17: 1545–1555. doi: 10.1364/JOSAA.17.001545. 10.1364/josaa.17.001545 10975364

[bibr63-2041669517739635] Webster M. A. Mollon J. D. (1995) Colour constancy influenced by contrast adaptation. Nature 373: 694–698. doi: 10.1038/373694a0. 7854451 10.1038/373694a0

[bibr64-2041669517739635] Weiss D. Bloj M. Gegenfurtner K. R. (under review) Perfect color constancy in a natural task and environment.

[bibr65-2041669517739635] Weiss D. Witzel C. Gegenfurtner K. R. (under review) Sensitivity to hue explains “blue bias” in perceived illumination.

[bibr66-2041669517739635] Winkler A. D. Spillmann L. Werner J. S. Webster M. A. (2015) Asymmetries in blue-yellow color perception and in the color of ‘the dress’. Current Biology 25: R547–R548. doi: 10.1016/j.cub.2015.05.004. 25981792 10.1016/j.cub.2015.05.004 PMC4489998

[bibr67-2041669517739635] Witzel C. (2016) New insights into the evolution of color terms or an effect of saturation? i-Perception 7: 1–4. doi: 10.1177/2041669516662040. 10.1177/2041669516662040 PMC5030748 27698987

[bibr68-2041669517739635] Witzel C. Cinotti F. O’Regan J. K. (2015) What determines the relationship between color naming, unique hues, and sensory singularities: Illuminations, surfaces, or photoreceptors? Journal of Vision 15: 19 doi: 10.1167/15.8.19. 10.1167/15.8.19 26114682

[bibr69-2041669517739635] Witzel C. Gegenfurtner K. R. (2013) Categorical sensitivity to color differences. Journal of Vision 13: 1 doi: 10.1167/13.7.1. 10.1167/13.7.1 23732118

[bibr70-2041669517739635] Witzel C. Gegenfurtner K. R. (2015) Chromatic contrast sensitivity. In: Luo R. (ed.) Encyclopedia of color science and technology, Berlin, Germany: Springer, pp. 1–7.

[bibr71-2041669517739635] Witzel, C., Hansen, T., & Gegenfurtner, K. R. (2008). *Wie sich Farben mit den Betrachtern und mit den Zeiten ändern*. [How colours change with observers and times]. Paper presented at the Tagung experimentell arbeitender Psychologen (TeaP), Marburg.

[bibr72-2041669517739635] Witzel C. Maule J. Franklin A. (2013) Focal colors as perceptual anchors of color categories. Journal of Vision 13: 1164 (VSS2013 abstracts.).

[bibr73-2041669517739635] Witzel C. Racey C. O’Regan J. K. (2017) The most reasonable explanation of “the dress”: Implicit assumptions about illumination. Journal of Vision 17: 1 doi: 10.1167/17.2.1. 10.1167/17.2.1 28146253

[bibr74-2041669517739635] Witzel C. Valkova H. Hansen T. Gegenfurtner K. R. (2011) Object knowledge modulates colour appearance. i-Perception 2: 13–49. doi: 10.1068/i0396. 23145224 10.1068/i0396 PMC3485772

[bibr75-2041669517739635] Witzel C. van Alphen C. Godau C. O’Regan J. K. (2016) Uncertainty of sensory signal explains variation of color constancy. Journal of Vision 16: 8 doi: 10.1167/16.15.8. 10.1167/16.15.8 27936272

[bibr76-2041669517739635] Wuerger S. M. Hurlbert A. C. Witzel C. (2015) Variation of subjective white-points along the daylight axis and the colour of the dress. Perception 44 (issue 1 supplement), 153.

[bibr77-2041669517739635] Wyszecki G. (1958) Evaluation of Metameric Colors. Journal of the Optical Society of America 48: 451–452. doi: 10.1364/JOSA.48.000451.

[bibr78-2041669517739635] Zaidi Q. Spehar B. DeBonet J. (1997) Color constancy in variegated scenes: role of low-level mechanisms in discounting illumination changes. Journal of the Optical Society of America. A, Optics, Image Science, and Vision 14: 2608–2621. doi: 10.1364/JOSAA.14.002608. 10.1364/josaa.14.002608 9316275

