# Supplementary material for: Determinants of Colour Constancy and the Blue Bias
Source: Iperception. 2017 Dec 6;8(6):2041669517739635. doi: 10.1177/2041669517739635 (PMC5768282; doi:10.1177/2041669517739635)
Supplement: Supplementary material [file supplementary_material.pdf]

Supplementary Material

Colour Naming Experiment

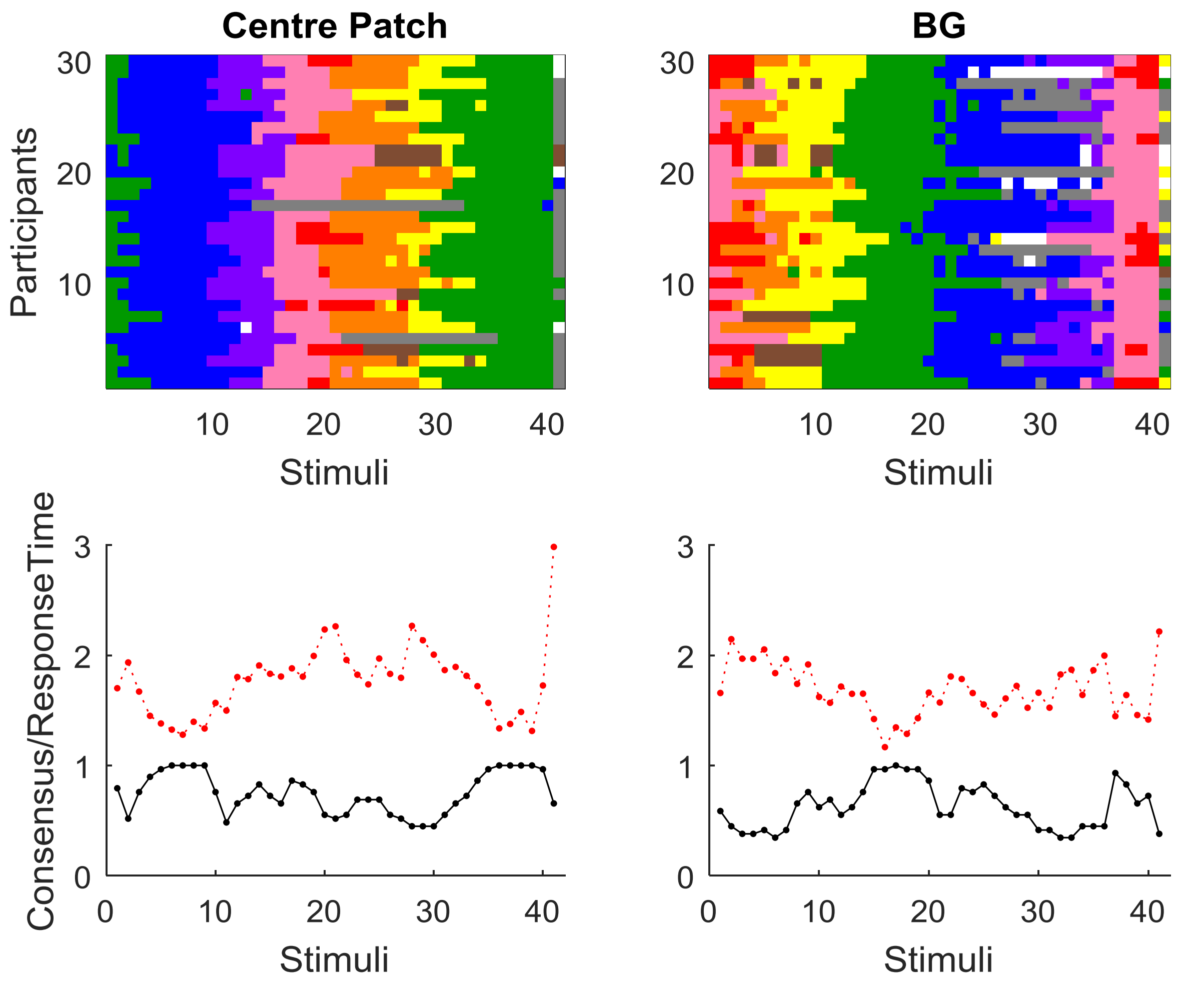

Figure S 1. Individual colour naming data. The black line in the lower graph indicates consistency, the dotted red line indicates response time.

DKL azimuth in degree	x	y	Y	Opponent Munsell Hue
213.8	0.287	0.338	46.61	2.5R
211.5	0.281	0.339	50.22	5R
218	0.281	0.333	49.21	7.5R
228.2	0.284	0.326	47.75	10R
234	0.284	0.321	48.49	2.5YR
240	0.286	0.315	48.32	5YR
243.6	0.286	0.310	50.18	7.5YR
250.2	0.289	0.306	49.29	10YR
254.4	0.293	0.303	48.50	2.5Y
257.2	0.295	0.302	47.77	5YR
260.3	0.295	0.297	50.66	7.5Y
263.7	0.299	0.298	48.67	10Y
266.3	0.301	0.295	48.69	2.5GY
269.2	0.303	0.291	49.75	5GY
281.3	0.315	0.293	48.67	7.5GY

292.2	0.325	0.294	49.77	10GY
315.2	0.344	0.298	52.83	2.5G
336.9	0.358	0.306	52.94	5G
351.4	0.367	0.312	52.89	7.5G
358.5	0.372	0.317	53.13	10G
8.400	0.376	0.325	53.28	2.5BG
18.60	0.380	0.333	53.59	5BG
24.70	0.385	0.339	53.40	7.5BG
29.70	0.382	0.345	52.46	10BG
36.50	0.378	0.350	51.62	2.5B
43.20	0.375	0.355	51.37	5B
45.60	0.373	0.358	51.83	7.5B
53	0.368	0.363	50.38	10B
59.90	0.365	0.367	51.77	2.5PB
65.10	0.360	0.370	49.98	5PB
76.80	0.355	0.377	52.44	7.5PB
83.70	0.349	0.380	49.85	10PB
91.30	0.343	0.383	48.77	2.5P
102.6	0.335	0.388	49.56	5P
119.1	0.322	0.395	51.11	7.5P
130.3	0.310	0.395	51.28	10P
145.2	0.299	0.388	51.01	2.5RP
163.9	0.290	0.375	51.81	5RP
179.8	0.286	0.363	50.83	7.5RP
193.3	0.283	0.352	51.95	10RP
NEUTRAL	0.327	0.342	48.70	N6.5

Table S1. Illuminant specifications. Judd-corrected chromaticity and luminance of all 41 illuminations.

Achromatic Adjustments

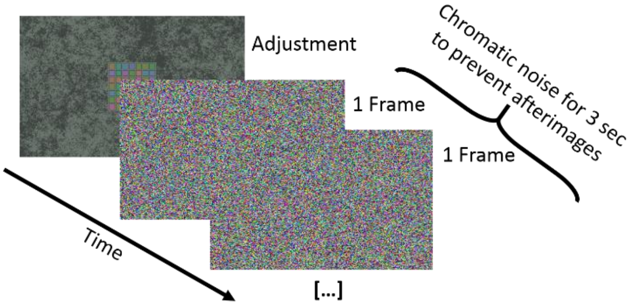

Figure S 2. A trial in the achromatic adjustment task. Note the chromatic noise patterns were presented after the adjustments to prevent after-images.

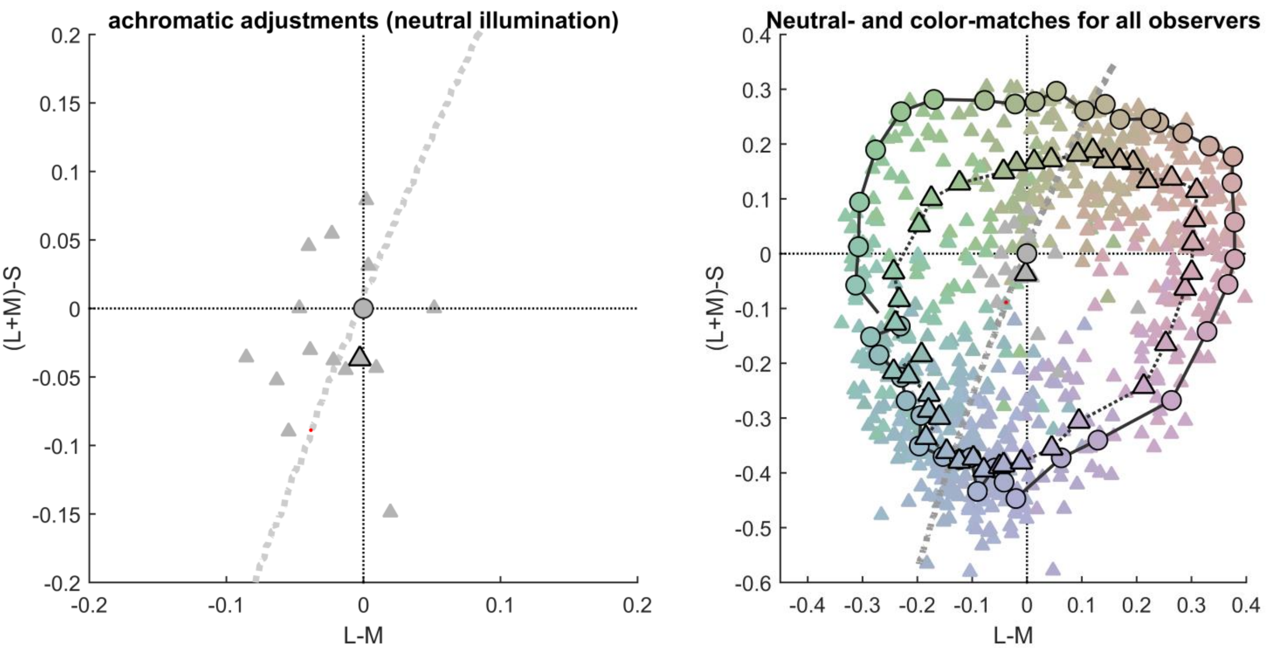

Figure S 3. A) Individual adjustments under the neutral illumination. Triangles depict neutral matches for 16 observers, the neutral illuminant is indicated by the circle with the back contour. The dotted line depicts the daylight locus from 5000K(top) to 8500K(bottom). Panel B shows individual adjustments(triangles) for all 41 illuminations tested in the experiment. Black-framed triangles depict average adjustments, black-framed discs indicate the coordinates of the illumination as measured from the brighter background surface.

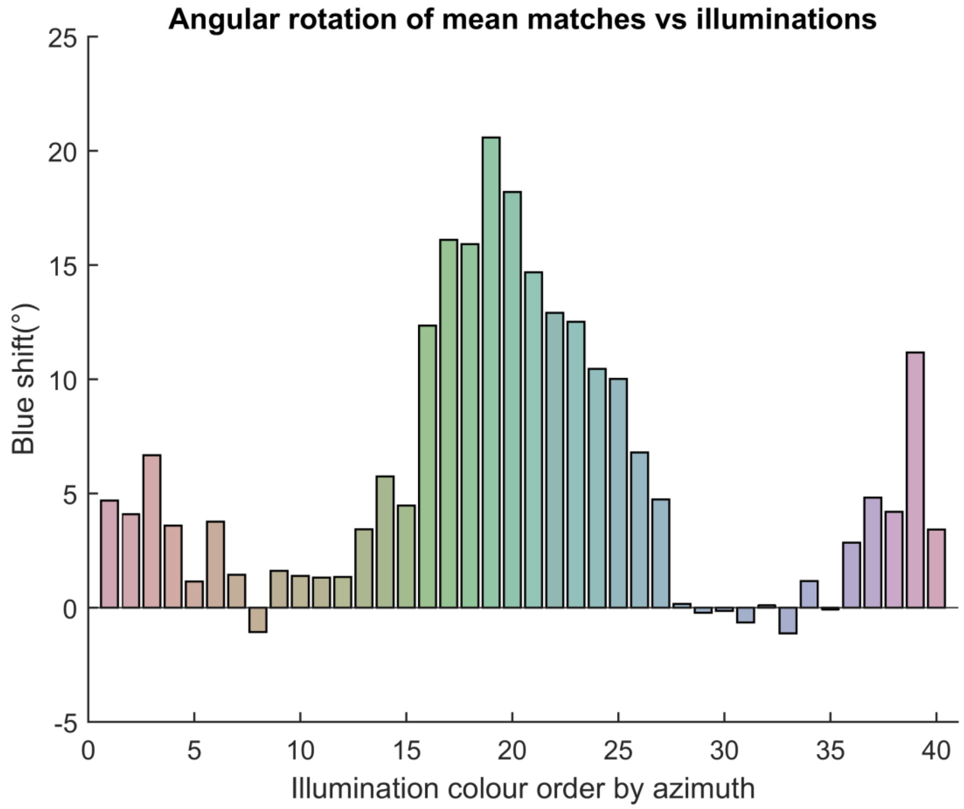

Figure S 4. Blue bias in angular rotations of mean matches relative to the according illuminations from yellow (78°) to daylight blue (238°). Positive values indicate that the average adjustment angle is shifted towards the angle of the bluish daylight illumination. The graphic demonstrates that the further illumination hues are away from the daylight blue–yellow axis, the more they are rotated towards blue.

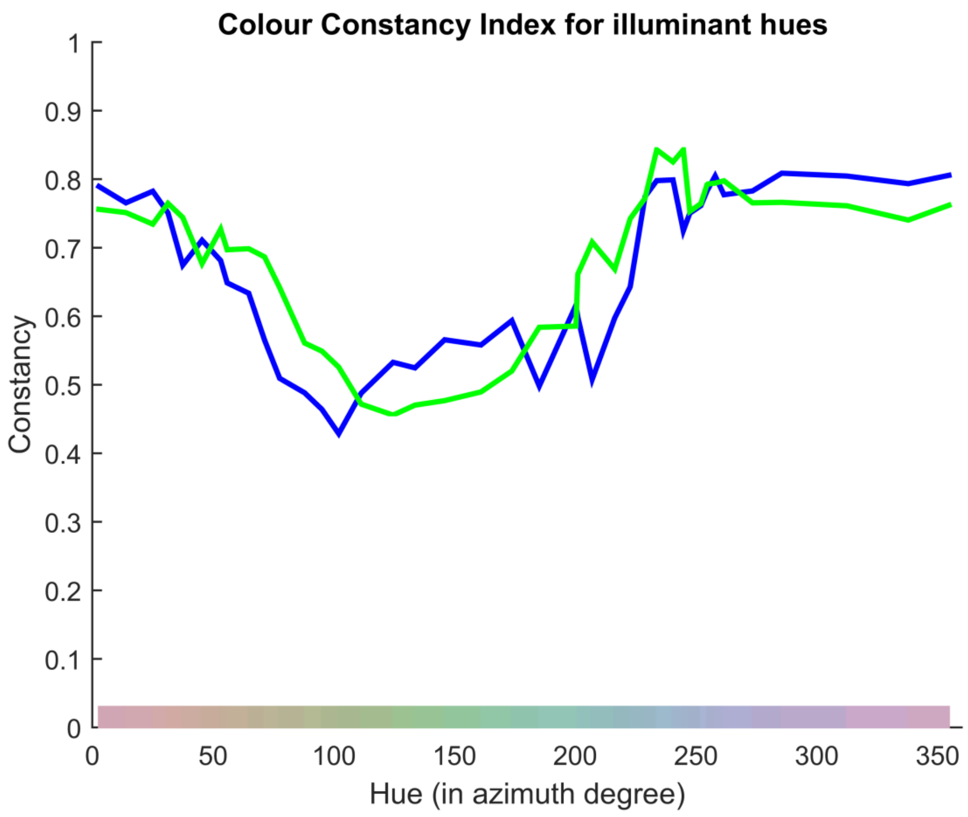

Figure S 5. Colour Constancy Index in DKL-space (green) and CIELAB (blue).

Comparison with illumination discrimination and chromatic detection

	df	Illumination discrimination		Chromatic Detection	
		r	p	r	p
CCI	10	-.003	.99	.08	.81
BR	10	.53	.08	.35	.26
Adj. error	10	.12	.71	-.04	.91
Interindi. Var.	10	.25	.43	.36	.25

Table S2. Correlations between illumination discrimination, chromatic detection, and colour constancy in DKL-space.

Candidate determinants

	df	Illumination shift		Blue bias		BG categories				Patch categories				Colour variegation			
						Consensus		RT		Consensus		RT		M		SD	
		r	p	r	p	r	p	r	p	r	p	r	p	r	p	r	P
CCI	38	.06	.71	**-.52**	**< .001**	**- .61**	**<.001**	**.42**	**.007**	.23	.15	-.3	.06	**.37**	**.02**	.21	.19
Adj. error	38	**.47**	**.002**	**.67**	**< .001**	**.73**	**<.001**	**-.54**	**<.001**	-.04	.78	.29	.07	**-.41**	**.008**	**-.40**	**.01**
Interindi. Var.	38	**.43**	**.005**	**.57**	**< .001**	**.70**	**<.001**	**-.53**	**<.001**	-.13	.42	**.38**	**.02**	**-.50**	**.001**	-.29	.07

	Metamer mismatch area				Sensory singularities				Cone ratio predictions			
	Correlations		Partial corr.		Correlations		Partial corr.		Correlation		Partial corr.	
	r	p	r	p	r	p	r	p	r	p	r	p
CCI	-.13	.43	**-.47**	**.002**	-.15	.35	-.09	.57	-.11	.5	-.26	.1
Adj. error	**.61**	**<.001**	**.51**	**< .001**	**.35**	**.03**	.12	.47	**.53**	**<.001**	.29	.07
Interindi. Var.	**.58**	**<.001**	**.52**	**< .001**	**.42**	**.008**	.22	.17	**.53**	**<.001**	**.35**	**.03**

Table S3. Correlations between colour constancy and candidate determinants in CIELAB. Bold faced numbers indicate significant correlations. Partial corr. = Partial correlations controlling for illumination shift (chroma of surfaces under neutral illumination in case of singularities.

Illumination shift

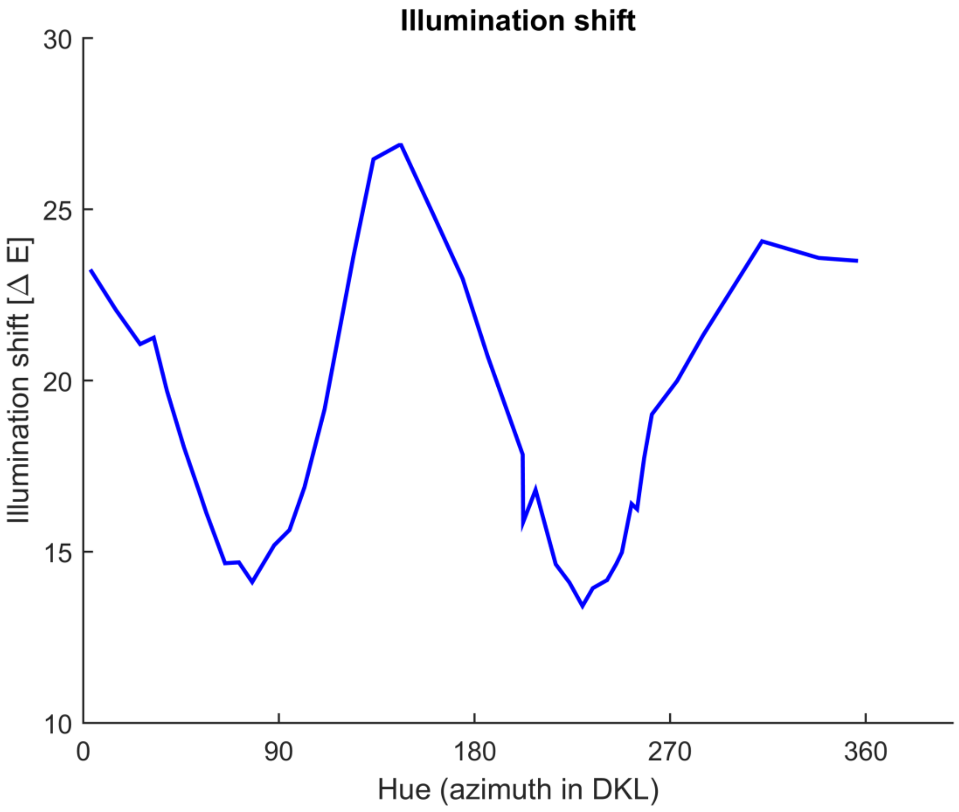

Figure S 6. Illumination shift in CIELAB.

Colour categories

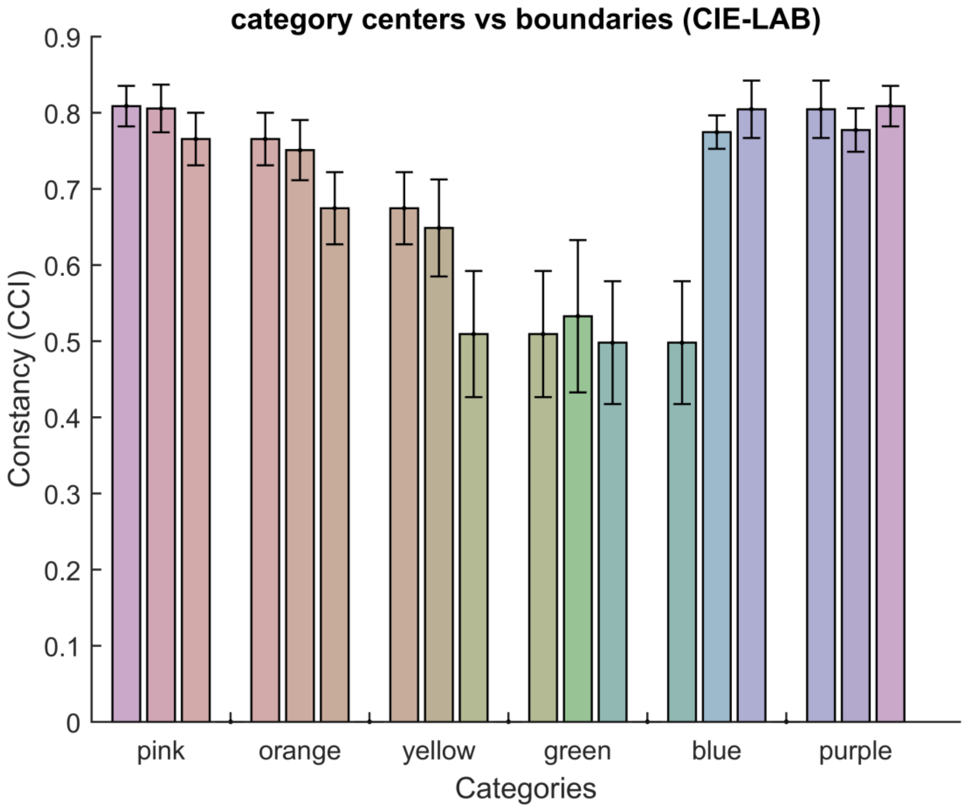

Figure S 7. Categorical colour constancy. The graphic compares the Colour Constancy Index between colour at the category centers and boundaries. Each group of three bars corresponds to one of the six categories. The bars in the center of each group correspond to the category centers, the other two to the category boundaries. The y-axis represents the Colour Constancy Index. In case of categorical colour constancy, the center colours (center bars) should yield higher constancy (higher bars) than the boundary colours. This is not the case for any of the categories.
